# GRAS-Di system facilitates high-density genetic map construction and QTL identification in recombinant inbred lines of the wheat progenitor *Aegilops tauschii*

**DOI:** 10.1038/s41598-020-78589-4

**Published:** 2020-12-08

**Authors:** Yuka Miki, Kentaro Yoshida, Hiroyuki Enoki, Shoya Komura, Kazuyo Suzuki, Minoru Inamori, Ryo Nishijima, Shigeo Takumi

**Affiliations:** 1 Graduate School of Agricultural Science, Kobe University, Rokkodai 1-1, Nada, Kobe, Japan; 2 toyota Motor Corporation, 1099, Marune, Kurozasa-cho, Miyoshi, Aichi Japan

**Keywords:** Plant sciences, Plant breeding

## Abstract

Due to large and complex genomes of Triticeae species, skim sequencing approaches have cost and analytical advantages for detecting genetic markers and building linkage maps. Here, we develop a high-density linkage map and identify quantitative trait loci (QTLs) for recombinant inbred lines of *Aegilops tauschii*, a D-genome donor of bread wheat, using the recently developed genotyping by Random Amplicon Sequencing-Direct (GRAS-Di) system, which facilitates skimming of the large and complicated genome and generates a large number of genetic markers. The deduced linkage groups based on the GRAS-Di genetic markers corresponded to the chromosome number of *Ae. tauschii.* We successfully identified stable QTLs for flowering time and spikelet shape-related traits. Genotype differences of RILs at the QTL-linked markers were significantly associated with the trait variations. In particular, one of the QTL-linked markers for flowering time was mapped close to *VRN3* (also known as *FLOWERING LOCUS T*), which controls flowering. The GRAS-Di system is, therefore, an efficient and useful application for genotyping and linkage mapping in species with large and complex genomes, such as Triticeae species.

## Introduction

Genotyping-by-sequencing methods such as whole-genome resequencing, RNA sequencing (RNA-seq), restriction site associated DNA sequencing (RAD-seq) and exome sequencing allow the construction of high-resolution linkage maps and identification of causal genes and quantitative trait loci (QTLs) for targeted phenotypes using mapping populations^1–4^. RNA-seq and RAD-seq are reduced-representation sequencing approaches with a relatively low cost, allowing genotyping of a large number of samples, even for species with large and complicated genomes^4^. These two approaches, however, had shortcomings compared with the whole-genome sequencing approach. Although RNA-seq can detect single nucleotide polymorphisms (SNPs) in expressed genes, expression variance among samples causes missing data. RAD-seq can cover SNPs in both coding and intergenic regions, but it often generates missing data in tested samples^3^. Therefore, imputation of missing data is widely used before genotyping. To overcome the shortcomings of both approaches, it is still important to develop another genotyping-by-sequencing method that facilitates both the acquisition of a large number of reproducible and high-confidence genetic markers at a low cost and the suppression of missing data.

**Figure 1:**
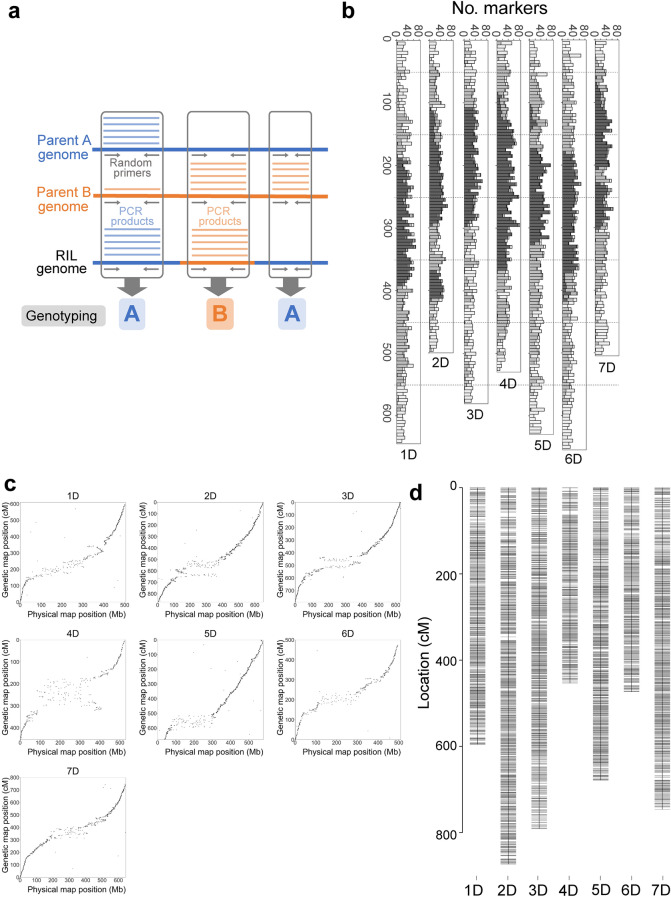
GRAS-Di markers in *Ae. tauschii* KU-2078/PI499262 RILs covered all the chromosomes and generated a genetic map with linkage groups corresponding to the seven chromosomes of *Ae. tauschii*. (**a**) Illustration of the genotyping method used for GRAS-Di markers. (**b**) Distribution of GRAS-Di markers on the *Ae. tauschii* physical map. Cumulative bar plots of the number of markers are shown every 10 Mbp. The markers were classified into three categories. The white category indicates 1–10 markers that shared the same segregating pattern of genotypes in the RILs. The grey category indicates 11 to 100 markers that shared the same segregating pattern of genotypes in the RILs. The dark grey category indicates more than 100 markers that shared the same segregating pattern of genotypes in the RILs. (**c**) Dot plots of the marker positions between the tau-D qABC genetic linkage and physical maps. The marker positions in the linkage map strongly corresponded to the physical map of *Ae. tauschii*. (**d**) A high-density linkage map of the RILs based on the tau-D qABC marker set. The x-axis represents the linkage group number, and the y-axis indicates genetic distance (cM). The erroneously mapped markers on the end of chromosome 5D and the large gaps were removed from the original tau-D qABC linkage map (Supplementary Fig. 3). The Cumulative bar plots and the dot plots were created with the R package ggplot2 (Wickham 2016). The linkage map was created with the R package R/qTL (Broman et al. 2003).

Genotyping by Random Amplicon Sequencing-Direct (GRAS-Di) is a recently developed method for genotyping by sequencing^5–7^. GRAS-Di is a derivative of amplicon sequencing technology and uses random primers for PCR amplification. GRAS-Di can identify a large number of genetic markers covering all chromosomes even among genetically similar individuals and can be applied to hundreds to thousands of samples by using primer sets containing different index sequences at a relatively low cost. Amplified regions are highly reproducible among technical replicates, making it possible to suppress missing data. GRAS-Di is applicable to species without reference genome sequences because the genotyping step of GRAS-Di can be conducted based on the presence and absence of amplified reads (Fig. 1a). When reference genome sequences are available, sequence information of the amplified reads is useful for anchoring to physical maps.

Triticeae is a botanical tribe including the important crops wheat and barley and contains species with large and complicated genomes. The genome size of hexaploid bread wheat is approximately 16 Gbp^8^. Even diploid Triticeae species have large genomes (~ 5 Gbp)^9–12^. Approximately 85% of the genome of these species is composed of transposable elements (TEs), increasing genome complexity^13^. To improve yield, tolerance to abiotic and biotic stresses, and grain quality, breeding and genetic analyses of Triticeae, including wild related species, have been conducted using mapping populations^14–16^. The development of efficient and inexpensive methods for genotyping is effective for accelerating the breeding and genetic analyses of Triticeae species. GRAS-Di is a potential method for meeting these demands. In the present study, to demonstrate the potential of GRAS-Di for genotyping, the wild diploid wheat *Aegilops tauschii* Coss. (formerly called *Ae. squarrosa* L.) was used as a representative species with a large and complicated genome.

*Aegilops tauschii* is the D-genome progenitor of common wheat and has a wide geographic distribution from Syria and Turkey to western China^17^. Large natural variation in spikelets, floral morphological traits and flowering time exist in *Ae. tauschii* populations^18–20^. Based on morphological differences in spikelets, two subspecies, namely, *Ae. tauschii* Coss. ssp. *strangulata* (Eig) Tzvel. and *Ae. tauschii* Coss. ssp. *tauschii*, were classified. *Ae. tauschii* ssp. *strangulata* forms quadrate spikelets, while the spikelets of *Ae. tauschii* ssp. *tauschii* are elongated and cylindrical^21,22^. Genome-wide DNA polymorphism analyses revealed that *Ae. tauschii* has two major divergent lineages, TauL1 and TauL2^23,24^. *Ae. tauschii* accessions belonging to TauL2 colonize relatively restricted habitats around Transcaucasia and northern Iran, whereas the TauL1 accessions are adapted to wide geographic regions from Syria and Turkey to western China. Given that *Ae. tauschii* ssp. *strangulata* is included in TauL2, morphological divergence is presumed to have occurred in TauL2 populations. A major causal QTL of the morphological divergence of spikelet shape-related traits between *Ae. tauschii* ssp. *strangulata* and *Ae. tauschii* ssp. *tauschii* was identified on chromosome 7D using an F_2_ mapping population derived from inter-subspecies crosses, suggesting that the QTL on chromosome 7D could be a major contributor to subspecies differentiation^25^.

The main purpose of the present study is to examine whether GRAS-Di is a powerful method for the detection of polymorphic markers, construction of linkage maps and identification of QTLs for species with large and complicated genomes. Recombination inbred lines (RILs) had been developed from F_2_ populations derived from a cross between *Ae. tauschii* ssp. *tauschii* PI499262 (TauL1b) and *Ae. tauschii* ssp. *strangulata* KU-2078 (TauL2), which were used in Nishijima et al. (2017). We applied GRAS-Di to these RILs, demonstrating that GRAS-Di efficiently captured a large number of genetic markers covering whole chromosomes and facilitated the construction of high-resolution linkage maps as well as the identification of QTLs for flowering time and spikelet-related traits. In particular, a potential causative gene of flowering time was successfully identified.

## Results

### GRAS-Di enables the attainment of a large number of genetic markers and the construction of a high-density linkage map

GRAS-Di of 96 RILs was performed to obtain an adequate number of genetic markers for constructing high-density linkage maps. An average of 4,070,273 paired-end reads per line were obtained, generating 1.229 Mbp data per line (Supplementary Table S1). The number of GRAS-Di markers was 78,198, of which 72,681 (92.9%) were polymorphic between the parental lines. The other markers were polymorphic in the RILs but were monomorphic between the parental lines. Given that markers should be polymorphic between the parental lines, these monomorphic markers were not used in the subsequent analyses. The GRAS-Di software ranks markers using A, B, C, D, and E in descending order of quality. Quality of GRAS-Di markers is empirically determined based on reproducibility of presence/absence of reads and the number of reads over samples in trial data of crop species. Markers of qualities D and E are less reliable than those of qualities A, B and C. Of the polymorphic markers, 48,460 (66.7%) were of quality A, B or C and were used for the downstream analyses (Supplementary Table S2).

By aligning forward and reverse reads of markers to the *Ae. tauschii* reference genome^10^, 30,614 markers were anchored to their chromosomes, resulting in 7.61 markers per Mbp (Supplementary Table S3). Of the total mapped read pairs, 98.5% showed that the insert size between forward and reverse reads was less than 300 bp (Supplementary Fig. S1). Since the insert size of the GRAS-Di library was 100 ~ 300 bp, markers whose distance between reads was over 300 bp were filtered out, generating 30,154 accurately mapped markers, of which 2119 (7.0%) were located in intragenic regions and 28,035 (93.0%) were located in intergenic regions. These markers covered all the chromosomes (Fig. 1b). If markers had identical segregating patterns of genotypes in RILs, these markers were counted as one locus, yielding 4521 loci (1.12 loci per Mbp) (Supplementary Table S4; Supplementary Data S1). The density of segregating loci increased towards the end of the chromosomes (Supplementary Fig. S1). Of the total loci, 54.2% (2451 loci) had one marker, 28.8% (1300 loci) had two to five markers per locus, and 17% (770 loci) had more than five markers per locus (Supplementary Fig. S1). Markers showing one-to-one relationships with loci were predominant at the end of chromosomes, while the loci with more than five markers were enriched in the proximal regions (Fig. 1b).

To examine which quality of GRAS-Di markers created high-density linkage maps, we generated five sets of GRAS-Di markers as described in Supplementary Fig. S2: all segregating markers of quality A or B (qAB); all segregating markers of quality A (qA); and markers showing one-to-one relationships with loci based on the *Ae. tauschii* genome and of quality A, B or C (tau-D qABC), of quality A or B (tau-D qAB), or of quality A (tau-D qA). Linkage maps based on each of the sets of markers exhibited seven linkage groups, which was consistent with the chromosome number of *Ae. tauschii* (Supplementary Fig. S3). The number of loci per cM was 0.68 to 0.96 (Supplementary Table S4). In all the linkage maps except that for tau-D qA, there were large genetic distances between neighbouring markers exceeding 200 cM. These abnormal genetic distances could be artefacts. The tau-D qABC linkage map showed the lowest log-likelihood (Supplementary Table S5). Although the tau-D qABC linkage map had a large gap at chromosome 7D, marker density in the tau-D qABC linkage map was higher than in the tau-D qA linkage map. The marker positions in the tau-D qABC linkage map corresponded to the physical map of *Ae. tauschii* (Fig. 1c). In the proximal regions, the correspondence between the two maps tended to be vague. The genetic distance between neighbouring markers exhibited small values, while the physical distance was large. Towards the end of the chromosome, genetic distance became more substantial than physical distance. The recombination rate (cM/Mb) was estimated over the chromosomes (Supplementary Fig. S4). The proximal regions tended to display a suppressed recombination rate, although there were some recombination hotspots.

The small portion at the end of chromosome 5D in the tau-D qABC linkage map was located to the opposite end of chromosome 5D in the physical map (Fig. 1c; Supplementary Fig. S3). This portion could be erroneously connected to chromosome 5D due to the lack of markers between the connected sides. Gaps were also found at the end of chromosomes 2D, 4D, and 7D. By removing these gaps and the portion that was incorrectly connected to chromosome 5D, a modified tau-D qABC linkage map was constructed (Fig. 1d) and used for further analyses.

### Phenotypic variations in KU-2078/PI499262 RILs

**Figure 2:**
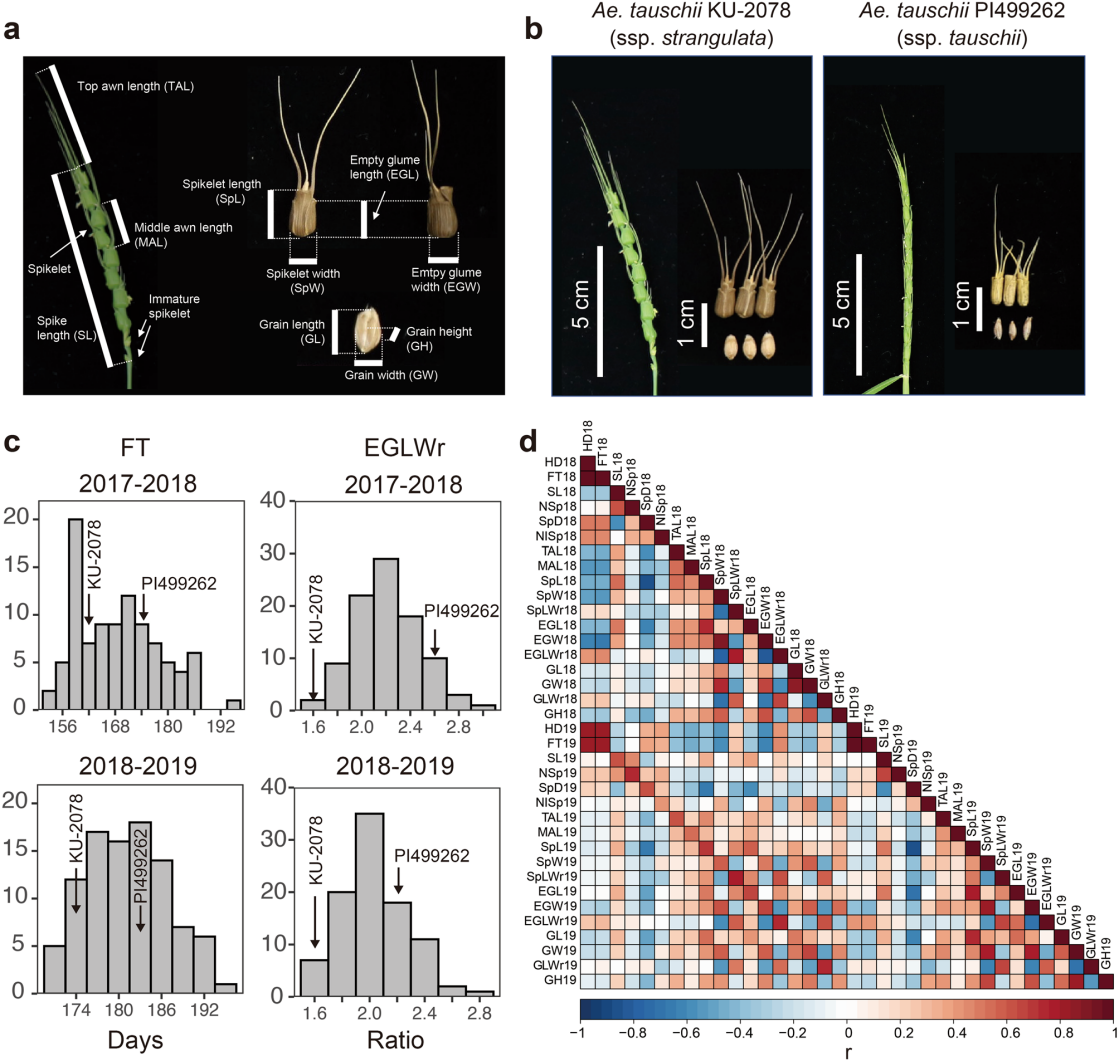
Evaluation of traits for *Ae. tauschii* KU-2078/PI499262 RILs and their parental accessions. (**a**) Measured spike, spikelet and grain traits. (**b**) Spike, spikelet and grain morphologies of the parental accessions. *Ae. tauschii* ssp. *strangulata* forms quadrate spikelets, while the spikelets of *Ae. tauschii* ssp. *tauschii* are elongated and cylindrical. (**c**) Histograms of flowering time (FT) and the empty glume length-to-width ratio (EGLWr) in Kobe, Japan, in the 2017–2018 and 2018–2019 seasons. (**d**) Pearson’s correlation coefficient (r) between the tested traits in the RILs in the two seasons. *HD* Heading date, *NSp* number of spikelets, *NISp* number of immature spikelets, *SpD* Spikelet density, *SpLWr* spikelet length-to-width ratio, *GLWr* grain length-to-width ratio. The histograms and the heatmap were created with the R packages ggplot2 (Wickham 2016) and corrplot (Wei and Simko 2017), respectively.

We assessed phenotypic variation in 16 morphological traits, flowering time (FT) and heading date (HD) for the RILs and their parental accessions KU-2078 and PI499262 in the two seasons 2017–2018 (2018) and 2018–2019 (2019) (Fig. 2ab; Supplementary Data S2). Compared with PI499262, KU-2078 had a larger number of spikelets (NSp) and earlier heading and flowering (Fig. 2c; Supplementary Fig. S5). The spikelet width (SpW), empty glume width (EGW), grain width (GW) and grain height (GH) of KU-2078 were clearly larger than those of PI499262. The spikelet length-to-width ratio (SpLWr), empty glume length-to-width ratio (EGWr), and grain length-to-width ratio (GLWr) of KU-2078 were smaller than those of PI499262. These results reflected the large morphological divergence between subspecies *tauschii* and *strangulata* (Fig. 2b). The distributions of morphological traits varied greatly between the seasons. Most of the traits followed normal distributions, supporting that QTLs governed these traits. On the other hand, some traits such as heading date in 2018 and 2019, grain length and grain width in 2018, and spike length in 2019 deviated from a normal distribution. The RILs showed widths, heights and length-to-width ratios of spikelet shape-related traits intermediate to those of the parental accessions, while they exhibited transgressive segregation in the other traits (Fig. 2c; Supplementary Fig. S5).

To examine correlations between the measured traits, pairwise Pearson’s correlation coefficients (r) were calculated (Fig. 2d; Supplementary Fig. S6). Heading date and flowering time showed highly positive correlation (r = 0.99 in 2017–2018 and r = 0.98 in 2018–2019), suggesting that these traits could be treated as the same traits. Flowering time had positive correlations with spikelet density and the number of immature spikelets but had negative correlations with awn length and spikelet shape-related traits such as spikelet and empty glume width. This result indicates that RILs with earlier heading and flowering tend to have reduced spikelet density and increased awn length and spikelet shape-related traits. Awn length was positively correlated with the sizes of spikelets, glumes and grains. There were also positive correlations between spike length and spikelet shape-related traits.

The reproducibility of trait values between years was confirmed based on Pearson’s correlation coefficient (Supplementary Fig. S7). Heading date, flowering time, and the number of spikelets exhibited high reproducibility between years (r ≥ 0.75). On the other hand, the other traits showed intermediate reproducibility (r = 0.42 to 0.71). Since the lengths of spikelets, empty glumes, and grains were positively correlated with their widths (Fig. 2d; Supplementary Fig. S6), these values should be normalized in comparisons between years. The spikelet length-to-width ratio, empty glume length-to-width ratio, and grain length-to-width ratio are presented as normalized values. Since these ratios showed correlation coefficients greater than 0.75, we used these ratios as representative values of spikelet shape-related traits for QTL analyses.

The less reproducibility of the traits between years suggests that environmental factors influenced phenotypic variations in the RILs. To examine contributions of genetic and environmental factors to the tested traits, genetic variance (V_g_), environmental variance (V_e_), variance of genotype by environment interaction (V_g x e_), residual variance (V_r_), and heritability (*h*) for each trait were calculated (Supplementary Table S6). *h* of the traits ranged from 0.15 to 0.63. Especially, *h* of the traits related to grain morphology was low. V_e_ of grain height, grain length, and grain width was larger than V_g_ of those. These results implied that environmental factors contributed to the observed phenotypic variations in the RILs. Some traits showed high V_r_, which might reflect technical errors or fewer replications of samples. The spikelet length-to-width ratio and empty glume length-to-width ratio had relatively high heritability.

### Identification of QTLs for flowering time and spikelet shape-related traits

**Figure 3:**
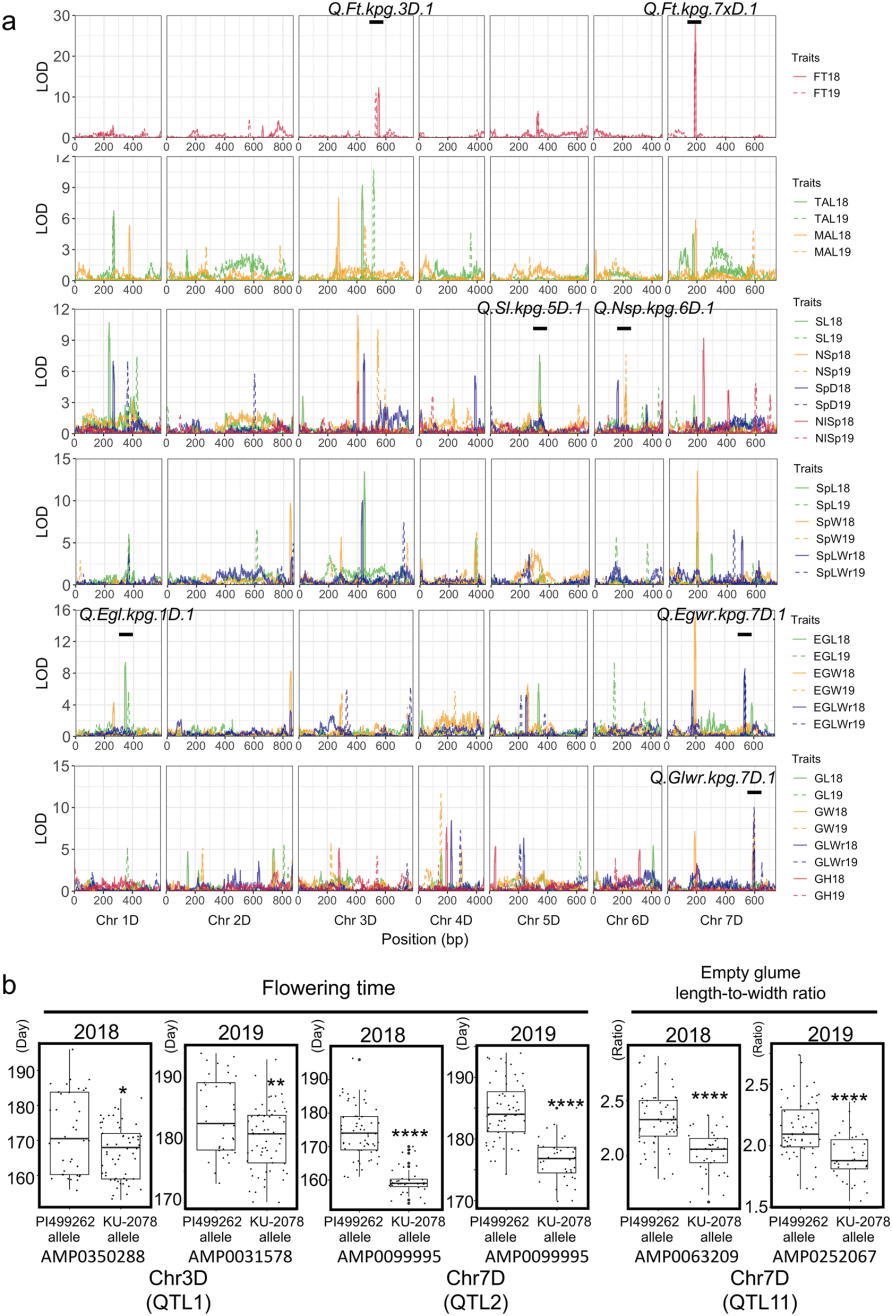
QTL analysis of *Ae. tauschii* KU-2078/PI499262 RILs. (**a**) Distribution of LOD scores for the 17 traits over the linkage map. The x-axis indicates the positions of the linkage map (cM), and the y-axis represents the LOD score. Positions of stable QTLs shown in Table 1 were marked as numbers one to seven on each peak. (**b**) Box and dot plots for flowering time and empty glume length-to-width ratio between RILs having the PI499262 allele and RILs having the KU-2078 allele at the QTL-linked markers in the 2017–2018 and 2018–2019 seasons. Welch's two-sample t-test was performed to assess statistical significance between the two genotypes of the RILs (*P < 0.05, **P < 0.01, ***P < 0.005, ****P < 0.001). Plots were created with the R package ggplot2 (Wickham 2016).

QTL analysis of the 17 traits was performed using the modified tau-D qABC linkage map. A logarithmic transformation of the spike length (SL) data was performed in advance to QTL analysis, resulting in near-normal distribution of SL. Given that significant LOD scores over the thresholds indicated the presence of QTLs (Supplementary Table S7), a genome scan of QTLs was conducted. Peaks of LOD scores in seven chromosomal regions involved in flowering time (FT), SL, number of spikelets (NSp), empty glume length (EGL), empty glume length-to-width ratio (EGLWr), and grain length-to-width (GLWr) were stable between years (Fig. 3a). The other peaks were detected only in one of the seasons. Since it was difficult to evaluate whether these peaks were caused by genetic, environmental, or artificial factors, we addressed only the reproducible QTLs. QTLs for flowering time were detected on chromosomes 3D and 7D (Table 1; Fig. 3a). Notably, the 7D QTL exhibited a significant and large LOD score (LOD score > 21). QTLs for spike length, number of spikelets, and empty glume length were detected on chromosomes 3D, 4D, and 5D, respectively. QTLs for EGLWr and GLWr were detected on chromosome 7D.

**Table 1:** Summary of LOD scores of the stable QTLs for traits in the two seasons.

Trait	QTL	Year	Chr	Positions on the genetic map (cM)	Positions on the physical map (bp)	Marker with the maximum LOD score	Position (cM)	Lod
FT	*Q.Ft.kpg.3D.1*	2018	3	546–555	1,403,169,549–98,699,078	AMP0350288	551	12.3***
		2019	3	527–536	1,596,656,621–114,361,083	AMP0031578	532	10.9***
	*Q.Ft.kpg.7D.1*	2018	7	185–194	630,168,46–74,799,744	AMP0099995	190	27.7***
		2019	7	185–194	630,168,46–74,799,744	AMP0099995	190	21.5***
SL	*Q.Sl.kpg.5D.1*	2018	5	333–343	421,264,059–363,347,606	AMP0045058	338	7.6*
		2019	5	340–350	429,259,117–363,347,606	AMP0194789	345	3.4
NSp	*Q.Nsp.kpg.6D.1*	2018	6	213–222	131,059,718–305,323,133	AMP0109251	218	4.8
		2019	6	207–219	131,059,718–315,351,308	AMP0109251	218	7.6*
EGL	*Q.Egl.kpg.1D.1*	2018	1	339–348	322,736,644–403,434,269	AMP0206621	344	9.3**
		2019	1	361–370	401,846,420–411,840,769	AMP0078930	366	5.7
EGWr	*Q.Egwr.kpg.7D.1*	2018	7	532–541	557,067,506–564,646,781	AMP0063209	537	8.1*
		2019	7	535–544	557,067,506–564,646,781	AMP0252067	538	8.5*
GLWr	*Q.Glwr.kpg.7D.1*	2018	7	590–600	592,738,365–599,236,655	AMP0092717	595	5.2
		2019	7	590–600	592,738,365–599,236,655	AMP0092717	595	9.9**

Positions of the physical map corresponded to positions of minimum and maximum bp of GRAS-Di markers that were located within QTLs (cM). Positions on the genetic maps included not only GRAS-Di markers but also markers that were estimated by the software r/qtl (Broman et al. 2003). The orders of some GRAS-Di markers on the physical map were not consistent with those on the genetic map. For these reasons, the length of physical positions of QTLs was not always corresponding to that of genetic positions. Positions on chromosomes 2, 3, 4, 5 on the genetic map were inversely ordered with those on the physical map. *P < 0.05, *P < 0.01, ***P < 0.001.

To clarify associations between traits and their QTL-linked markers, the distribution of trait values for genotypes of RILs at each of these linked markers was estimated (Fig. 3b; Supplementary Fig. S8). The trait comparisons between KU-2078 and PI499262 genotypes at the QTL-linked markers gave statistically significant results, indicating associations between traits and their QTL-linked markers. KU-2078 genotypes for these markers accelerated flowering and decreased EGLWr, while PI499262 genotypes delayed flowering and increased EGLWr.

The effects of combinations of QTL alleles on traits were also evaluated (Supplementary Fig. S9). For flowering time, the 7D QTL more strongly influenced phenotypes than the 3D QTL. The combination of PI499262 genotypes at both QTLs led to a significant delay compared with that observed with only one PI499262 genotype at one of the QTLs.

Considering that flowering time had a negative correlation with empty glume width (Fig. 2d; Supplementary Fig. S6), an interaction between QTLs for these traits potentially exists. To test this possibility, we focused on 7D QTLs and examined the interaction between the QTLs for flowering time and EGLWr (Supplementary Fig. S10) because the 7D QTLs of these traits were stably detected in the same region in the two seasons. Neither genotype at the 7D QTL for EGLWr influenced flowering time. On the other hand, EGLWr was dependent on genotypes at the 7D QTL for flowering time. When both of the QTLs were KU-2078 genotypes, EGLWr tended to decrease. Empty glume width was evaluated based on the genotypes of the marker linked to EGLWr. When QTLs for flowering time and EGLWr had KU-2078 genotypes, empty glume width tended to increase, especially in 2018.

### Annotated genes located around the detected QTLs

Genes located near the 3D and 7D QTL-linked markers were estimated using the gene model of *Ae. tauschii* (Supplementary Table S8). The 3D QTL region involved in flowering time includes a MADS box transcription factor, cytokinin dehydrogenase 2-like, *RICE FLOWERING LOCUS T 1*-like (RICE *FT1*-like), and a *PCF5*-like transcription factor. MADS box transcription factors such as *AGAMOUS* are homeotic genes involved in inflorescence formation and may affect the morphology of flower organs^26^. Cytokinin oxidase/dehydrogenase 2-like is an enzyme that degrades the plant hormone cytokinin, and it is known that mutants of *OsCKX2* (*Gna1*) increase the number of spikelets and plant height in rice^27^. *RICE FT1* is a homologue of *Hd3a*. *Hd3a* acts as a rice florigen under long-day conditions^28,29^. Therefore, the homologue of *RICE FT1* in this region might affect the flowering time of RILs. *PCF5* is a transcription factor belonging to class II of the TCP family. It is known that transcription factors with a TCP domain act on the whole plant morphology, such as leaf and flower shapes^30^. *TEOSINTE BRANCH1* (*TB1*), a TCP transcription factor, has been shown to interact with *FT1* in bread wheat and affect spikelet morphology^31^.

*VRN3* is located close to the flowering time QTL on chromosome 7D. *VRN3* is associated with the vernalization requirement in wheat and barley and is an important gene involved in the control of flowering^32–34^. In addition, the AP2-like ethylene-responsive transcription factor *AIL5*, which is involved in floral development^35^, and a gene encoding MADs transcription factor were located near the 7D QTL for GLWr and EGLWr, respectively.

**Figure 4:**
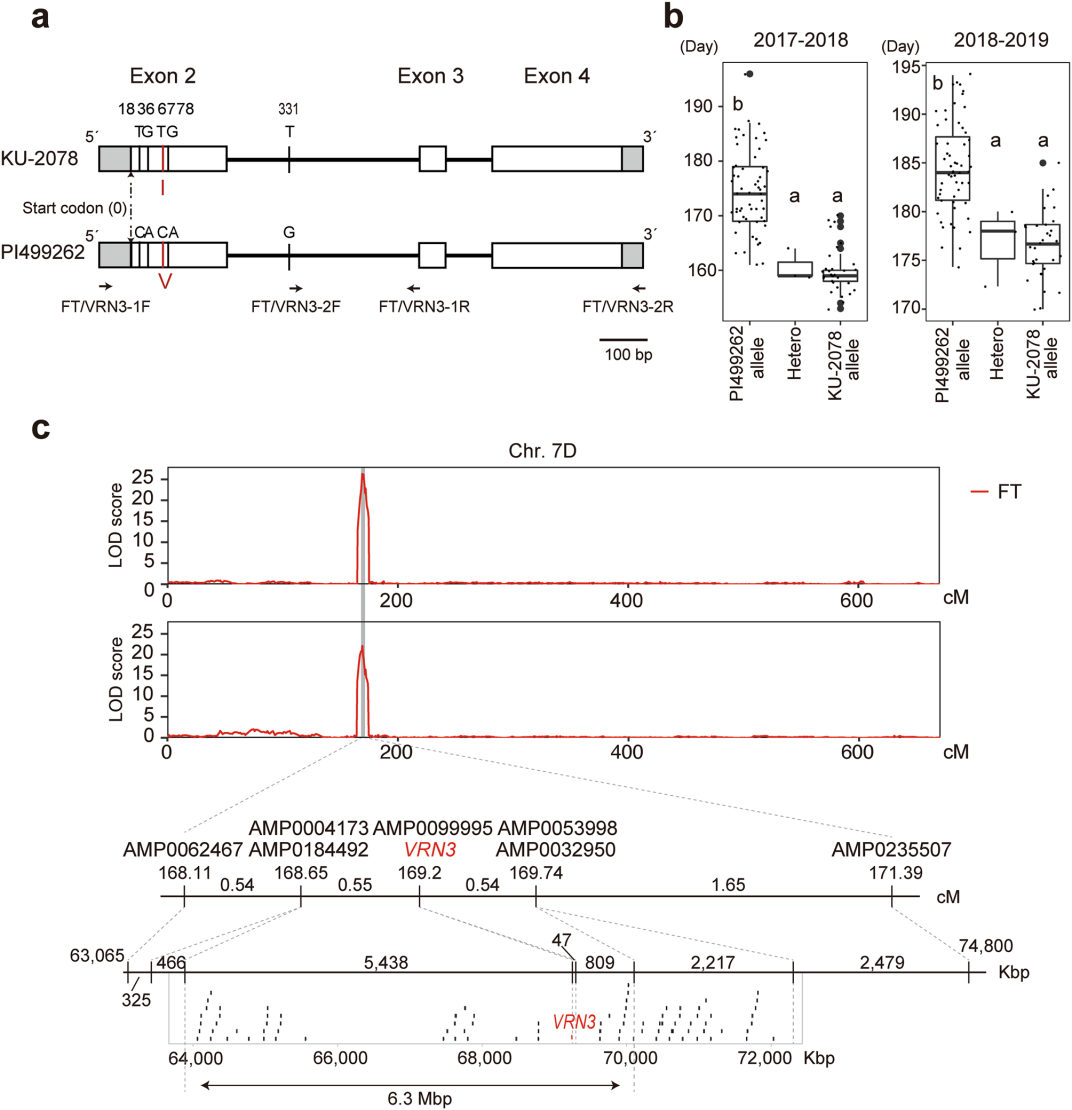
The wheat flowering locus *VRN3* was linked to the 7D QTL marker with the highest LOD score for flowering time. (**a**) Polymorphic sites in *VRN3* between the parental accessions KU-2078 and PI499262 of *Ae. tauschii*. White boxes are exons. Grey boxes are UTRs. Vertical bars indicate polymorphic sites. The red bar indicates the nonsynonymous substitution designed for the CAPS marker. The sites of primers for sequencing and the CAPS marker are shown with black arrows. (**b**) Box and dot plots of flowering time between the different genotypes (PI499262/PI499262 homozygous allele, PI499262/KU-2078 heterozygous allele, and KU-2078/KU-2078 homozygous allele) at *VRN3* in the *Ae. tauschii* KU-2078/PI499262 RILs in the 2017–2018 and 2018–2019 seasons. The same letters indicate no significant difference (P > 0.05, Tukey–Kramer HSD test). (**c**) Distribution of LOD scores for flowering time (FT) over the linkage map corresponding to chromosome 7D after removal of the RILs with the heterozygous genotype at *VRN3*. Enlarged view of the linkage map around *VRN3* and its corresponding physical map are shown below the distribution of LOD scores. Locations of genes between two markers, AMP004173 and AMP0053998, are shown as black and red dots below the physical map. The red dot is the location of *VRN3*. Plots were created with the R packages ggplot2 (Wickham 2016) and GenomicFeatures (Lawrence et al. 2013).

Comparison of coding sequences of *VRN3* between the parental accessions (KU-2078 and PI499262) revealed four nucleotide differences, with the one at the 67th position of *VRN3* causing an amino acid substitution from isoleucine to valine (Fig. 4a). A CAPS marker, which distinguished between KU-2078 and PI499262, was constructed based on the substitution at the 67th position (Supplementary Fig. S11). Homozygous and heterozygous KU-2078 genotypes of *VRN3* were associated with significantly earlier flowering than homozygous PI499262 genotypes (Fig. 4b). A linkage map was reconstructed using the RILs after excluding those with the heterozygous genotype for *VRN3.* The regions of *VRN3* and AMP0099995 were identical, with the highest peak LOD score for flowering time (Fig. 4c). The genetic distance between *VRN3* and AMP0099995 was 0 cM. *VRN3* and AMP0099995 were flanked by AMP004173 and AMP0032950, spanning 6.3 Mbp and containing 54 genes.

## Discussion

GRAS-Di generated a large number of genetic markers distributed over all the chromosomes and allowed the construction of high-resolution linkage maps. With the GRAS-Di genotype platform, genetic markers are categorized into five ranks, A, B, C, D and E, in descending order of genotyping reliability. In the present study, genetic markers were additionally classified into mapped and unmapped markers based on alignments to the *Ae. tauschii* reference genome. We performed linkage analyses of the 96 RILs using the six sets of genetic makers consisting of various combinations of these categorized genetic markers. All the sets of genetic markers created linkage maps consisting of seven linkage groups corresponding to the chromosomes of *Ae. tauschii*. This result indicates that GRAS-Di has the capacity to generate enough genetic markers to form complete linkage groups even if reference genome sequences are not available and the number of RILs is relatively small. In addition, without imputation, 48,460 segregating markers were obtained in the RILs of *Ae. tauschii*. Given that most existing methods, such as RAD-seq and RNA-seq, generate missing data and usually involve performing imputation after genotyping^3^, GRAS-Di has a considerable advantage in its exiguous missing data. These segregating markers were derived from both intragenic (7.0% of mapped markers) and intergenic (93.0% of mapped markers) regions. Considering that the percentage of the total length of the high-confidence-class genes in the *Ae. tauschii* genome is 7.5%^10^, GRAS-Di can detect markers in proportion to the ratio of intra- to intergenic regions in the genome. This characteristic makes it feasible to perform genome-wide genotyping.

In the present study, although the total length of linkage maps depended on the six sets of genetic markers, it was relatively long in all the maps. This could be partially explained by the high density of genetic markers, which generally increased the total length of the linkage map compared with that obtained with a low density of genetic markers due to the different powers to detect double crossing-over events. Another factor is the existence of large gaps in each linkage map. Although the chromosomal positions of most markers corresponded to those of the physical map of *Ae. tauschii*^10^, some markers exhibited inconsistencies in the positional relationship between the linkage and physical maps (Fig. 1d). These irregular markers generated genetic distances between two neighbouring markers with large values exceeding 200 cM (Supplementary Fig. S3).

Which factors caused the inconsistencies between the genetic linkage and physical maps? Given that the genotyping step of GRAS-Di is based on the presence and absence of reads for each RIL, GRAS-Di markers are regarded as dominant markers. If RILs contain heterozygous regions, genotypes at heterozygous positions are the same as those at homozygous sites where reads are present (Fig. 1a). Such erroneous genotyping at heterozygous regions could cause an inconsistency in marker positions between linkage and physical maps. Additionally, alignment errors of marker reads could result in the inconsistency (Fig. 1d). The genome of *Ae. tauschii* contains over 80% transposable elements^10^, indicating that many repetitive and similar nucleotide sequences exist in the *Ae. tauschii* genome. Of the segregating markers, 97% were located in intergenic regions. Dot plots between positions of genetic and physical maps showed that the correspondence of marker positions between the maps was vague in the proximal regions with more repeats, implying the existence of alignment errors. In addition, considering that relatively short marker sequences of 50 to 99 bp (average: 95.6 bp) were aligned to the *Ae. tauschii* reference genome, there might be markers whose alignment positions could be different from their genuine positions.

Of the segregating markers, 45.8% of loci had more than one marker showing the same genotyping pattern (Supplementary Fig. S1), which were mostly distributed in the proximal regions of the chromosomes on the physical map (Fig. 1b). The recombination rate was low around the chromosomal locations of these markers (Supplementary Fig. S4), suggesting that the suppression of recombination caused the same genotyping pattern. This pattern was consistent with that reported in previous studies of the wheat genome based on other genotyping platforms, in which recombination infrequently occurred in the proximal region and the recombination rate tended to increase towards the distal region of chromosomes^10,11,36^. In other words, the detection of many markers with the same genotyping pattern proved that GRAS-Di has the potential to obtain more segregating markers if it is applied to mapping populations with a larger size.

QTL analyses with GRAS-Di markers identified QTLs for flowering time, which were supported by high LOD scores. QTLs for flowering time were detected on chromosomes 3D and 7D. The 7D QTL exhibited a higher LOD value than the QTL on chromosome 3D, exerting a more considerable influence on flowering time. The florigen gene *VRN3*, which controls flowering in barley and wheat^32–34^, is located in the position corresponding to the peak of the 7D QTL. Given that the SNP marker discriminating the parental genotypes within *VRN3* was completely linked to the QTL on chromosome 7D (Fig. 4), *VRN3* is likely to be a major causal gene of differential flowering time in the RILs of *Ae. tauschii*.

Spikelet and seed shapes are characteristic traits that distinguish between subspecies. The GRAS-Di approach identified QTLs involved in diversification into subspecies on chromosome 7D. The detected chromosome of QTLs for the spikelet shape-related traits was consistent with those reported in previous studies using F_2_ populations of *Ae. tauschii*, although the chromosomal locations of the QTLs were different among studies^25,37^. This difference in the locations of QTLs could be caused by the usage of different parental accessions of *Ae. tauschii* and differences in environmental conditions. In the present study, QTLs for EGLWr and GLWr were stably detected on chromosome 7D (Fig. 3a), although most of the QTL regions of these spikelet shape-related traits were not stable between the two seasons. Evaluations of phenotypic variations in RILs revealed significant correlations between flowering time and spikelet shape-related traits (Fig. 2d). One of the genes near the QTL, *TB1,* is involved not only in the development of spikelet morphology but also in flowering by interacting with FT protein^31^. The observed correlation between flowering and spikelet morphology might be explained by interactions between QTLs for flowering time and spikelet shape-related traits. In fact, the 7D QTL for flowering time influenced EGLWr and empty glume width. On the other hand, the 7D QTL for the EGLWr did not affect flowering time (Supplementary Fig. S10). This unidirectional interaction suggests that causative genes for spikelet shape-related traits at the 7D QTL could be downstream of flowering genes such as *VRN3,* which was close to the marker with the highest LOD score for flowering time.

## Methods

### Plant materials

RILs (KU-2078/PI492262) were used for genotyping with GRAS-Di, evaluating phenotypic traits and QTL analysis. The parental accessions of the RILs are *Ae. tauschii* ssp. *strangulata* KU-2078 and *Ae. tauschii* ssp. *tauschii* PI499262. KU-2078 and PI492262 belong to the sublineages TauL1 and TauL2, respectively. In the 2017–2018 season, 96 individuals (F_8_ generation) were used for the evaluation of phenotypic traits and construction of GRAS-Di libraries. In the 2017–2018 season, 95 individuals (F_9_ generation) were used for the evaluation of phenotypic traits. These RIL individuals were grown from early November to early June in each of the seasons in the field of Kobe University, Nada-Ku, Kobe (N34.7°, E135.2°).

### Evaluation of phenotypic traits for the RILs

Heading date (HD), flowering time (FT), spike length (SL), number of spikelets (NSp), number of immature spikelets (NISp), top of awn length (TAL), middle of awn length (MAL), spikelet length (SpL), spikelet width (SpW), empty glume length (EGL), empty glume width (EGW), grain length (GL), grain width (GW), and grain height (GH) were measured for 98 RILs and their parental accessions KU-2078 and PI499262 (Fig. 2ab; Supplementary Data S2). Spikelet density (SpD) was calculated as Nsp per 1 cm SpL. The ratio of SpL to SpW (spikelet length-to-width ratio: SpLWr), ratio of EGL to EGW (empty glume length-to-width ratio: EGLWr), and ratio of GL to GW (grain length-to-width ratio: GLWr) were calculated. Since HD and FT were highly correlated, only FT was focused for the subsequent QTL analyses. The grain shape-related traits of 10 seeds per individual in 2018 and 15 seeds per individual were measured using *SmartGrain* software version 1.2^38^. When more than one seed per spikelet was observed, the seed with the largest size was selected for measuring grain shape-related traits. Three replicates per individual were used to measure the traits, excluding the above grain shape-related traits. Estimation of Pearson’s correlation coefficient and Welch’s t-test were conducted using R software ver. 3.3.1 (https://www.R-project.org/). The heatmap of correlation between the traits was created with the R package corrplot^39^. Maximum likelihood estimates of V_g_, V_e_, V_g x e_, and V_r_ for each trait in linear mixed-effects models were calculated using lmer function in the lme4 package for R software^40^. Heritability (*h*) was calculated based on the formula V_g_ /( V_g_ + V_e_ + V_g x e_ + V_r_).

### GRAS-Di analysis

DNA was extracted from mature leaves of 96 four-month-old individuals (F_8_ generation) and the two parental accessions that were grown in the field by using the CTAB method. GRAS-Di libraries were constructed according to the protocol described in Hosoya et al.^7^. Sequencing of the libraries was conducted using the Illumina HiSeq series. Genotyping was performed using GRAS-Di software (TOYOTA, Aichi, Japan), which is commercially available. The software evaluates marker quality according to the empirical criteria of genotyping reproducibility that is determined based on the number of reads and reproducibility of genotyping (presence and absence of reads) over samples by using trial GRAS-Di data of crop species (Patent ID P2018-42548A), and ranks markers using A, B, C, and D in descending order of reproducibility (A: reproducibility ≥ 99.99%, B: 99.98% ≤ reproducibility < 99.99%, C: 99.9% ≤ reproducibility < 99.98%, and D: 99.8% ≤ reproducibility < 99.9%). E-ranked markers contain tested samples with missing values. A-, B-, C-ranked markers are of high quality and can be used for genotyping. D-ranked markers are of sufficient quality and can be used for genotyping. E-ranked markers are not recommended for genotyping.

To choose which filtering options are effective for genotyping, five sets of filtered markers, qAB, qA, tau-D qABC, tau-D qAB and tau-D qA, were prepared according to the pipelines shown in Supplementary Fig. S2. Paired-end short reads of markers were aligned to *Ae. tauschii* reference genome v4.0^10^ using BWA-MEM version 0.7.12^41^. Mapped paired reads with a mapping quality score ≥ 40, which was calculated in BWA-MEM and is equal to about 0.01% probability that a read was misplaced, were obtained by using SAMtools version 1.9^42^. We selected mapped markers with distances between their forward and reverse reads < 300 bp. The marker distribution over the chromosomes was visualized using the ggplot2 package^43^ in R software ver. 3.3.1.

### Construction of linkage maps and QTL analysis

If markers had identical segregating patterns of genotypes in the RILs, these markers were used as one locus to remove marker redundancy. Linkage maps were constructed based on non-redundant markers (loci) using the R package OneMap^44^. According to the instructions of OneMap, two-point recombination fractions were estimated under a maximum recombination fraction < 0.5. Logarithm of the odds (LOD) score thresholds for statistical significance in two-point tests for linkage between markers were calculated using the “suggest_lod” function. After markers were assigned to linkage groups, genetic mapping of linkage groups was performed using the Kosambi mapping function.

QTLs were identified by using the R package R/qtl^45^. The type of population was set to RIL. Conditional genotype probabilities were calculated using the “calc.genoprob” function. The maximum distance between positions where the genotype probability was calculated was set to 1 cM. Composite interval mapping was conducted based on Haley-Knott regression. Permutation tests were conducted to identify markers with statistically significant LOD scores. The number of permutation replicates and the number of marker covariates were 1000 and five, respectively. A chromosomal region corresponding to markers/loci with a significant LOD score (p-value < 0.05) was regarded as a QTL. Dot plots between positions of genetic and physical maps, box and dot plots of traits for genotypes, and a diagram of gene locations were created using the ggplot2^43^ and GenomicFeatures^46^ packages in R software ver. 3.3.1. Tukey–Kramer HSD tests were performed using R software ver. 3.3.1.

### Identification of genes located near QTLs and construction of the CAPS marker of *VRN3*

To estimate the function of genes located near QTLs, *Ae. tauschii* high-confidence gene models (*Ae. tauschii* reference genome v4.0) were used as the query for BLASTP against the NCBI non-redundant protein database to search functional annotations. To amplify the *VRN3* region of *Ae. tauschii*, two primer pairs, 5´-CTGCTGCTTGCTCCCTCGTA-3´ (FT/VRN3-1F) and 5´- GCAGTACACGCGTGCACATC-3´ (FT/VRN3-1R) and 5´-TGTTTGTCTTGGCAGGCACA-3´ (FT/VRN3-2F) and 5´- AATTTGCTGACTTGGCGGCG-3´ (FT/VRN3-2R), were used (Fig. 4a). PCR amplification of *VRN3* was conducted using ExTaq polymerase (Takara Bio, Shiga, Japan) under the following conditions: 1 min at 94 °C for pre-denaturing, 40 cycles of 30 s at 94 °C, 30 s at 58, and 45 s at 68 °C, and 1 min at 72 °C for post-extension. A BigDye Terminator Cycle Sequencing Kit (Applied Biosystems, Foster City, CA, USA) was used to sequence the purified PCR products. Sequencing was performed using an Applied Biosystems 3730xl DNA Analyzer (Applied Biosystems).

Total DNA was extracted from leaves of the RILs and the parental accessions using the CTAB method. FT/VRN3-1F and FT/VRN3-1R primers were used for CAPS markers of *VRN3*. PCR amplification of the 0.6 kbp *VRN3* fragment was conducted using Quick Taq HS DyeMix (TOYOBO, Osaka, Japan) under the following conditions: 40 cycles of 10 s at 94 °C, 30 s at 58 °C, and 45 s at 68 °C. The fragments digested with *Fok*I were separated by 2% agarose gel electrophoresis and visualized under UV light after staining with ethidium bromide.

## Supplementary Information

Supplementary Information 1.

Supplementary Information 2.

## Data Availability

All data generated or analysed during this study are included in this published article as supplementary data and in the DDBJ Sequence Read Archive under accession numbers DRA010157. The accession number of the coding sequences of *VRN3* of *Ae. tauschii* PI499262 and KU-2078 was LC545559 and LC545560, respectively. Raw data for GRAS-Di analysis have been deposited at https://github.com/PlantGeneticsKobeU/GRAS-Di_RILs_Ae_tauschii/.
