# Supplementary material for: GRAS-Di system facilitates high-density genetic map construction and QTL identification in recombinant inbred lines of the wheat progenitor Aegilops tauschii
Source: Sci Rep. 2020 Dec 8;10:21455. doi: 10.1038/s41598-020-78589-4 (PMC7723059; doi:10.1038/s41598-020-78589-4)
Supplement: Supplementary file 1 — Supplementary Information 1. [file 41598_2020_78589_MOESM1_ESM.pdf]

Supplementary information

GRAS-Di system facilitates high-density genetic map construction and QTL identification in recombinant inbred lines of the wheat progenitor *Aegilops tauschii*

Yuka Miki[1], Kentaro Yoshida[1*], Hiroyuki Enoki[2], Shoya Komura[1], Kazuyo Suzuki[2], Minoru Inamori[2], Ryo Nishijima[1], Shigeo Takumi[1]

1. Graduate School of Agricultural Science, Kobe University, Rokkodai 1-1, Nada, Kobe, Japan

2. TOYOTA MOTOR CORPORATION, 1099, Marune, Kurozasa-cho, Miyoshi, Aichi, Japan

*Address correspondence to;

Kentaro Yoshida

Graduate School of Agricultural Science, Kobe University

Rokkodai 1-1, Nada, Kobe, Japan

Tel: +81-78-803-5858

E-mail: kentaro.yoshida@port.kobe-u.ac.jp

Supplementary Table S1 Sequencing results of the GRAS-Di library in the KU-2078/PI499262 RILs of *Ae. tauschii.*

RILs	Generation	Reads	Yield (Mbp)	Mean Quality Score	% Bases ≥ 30
#1	F_8	3,900,219	1,178	37.87	90.59
#2	F_8	4,356,995	1,316	37.92	90.75
#3	F_7	4,181,593	1,263	37.82	90.42
#4	F_8	4,219,043	1,274	37.93	90.81
#5	F_6	4,439,560	1,341	37.79	90.32
#6	F_7	4,084,538	1,234	37.87	90.57
#7	F_8	4,019,476	1,214	37.88	90.62
#8	F_8	4,427,493	1,337	37.85	90.52
#9	F_8	4,487,712	1,355	37.89	90.67
#10	F_5	4,098,965	1,238	37.89	90.66
#11	F_8	4,190,525	1,266	37.97	90.96
#12	F_8	4,130,884	1,248	37.94	90.82
#13	F_8	4,155,912	1,255	37.94	90.86
#14	F_8	4,363,544	1,318	37.94	90.86
#15	F_8	4,272,328	1,290	37.92	90.77
#16	F_8	4,339,466	1,311	37.98	90.97
#17	F_8	4,144,672	1,252	37.81	90.4
#18	F_8	4,603,278	1,390	37.95	90.85
#19	F_8	3,978,897	1,202	37.89	90.66
#20	F_8	3,892,850	1,176	37.95	90.86
#21	F_8	4,127,810	1,247	37.89	90.66
#22	F_8	4,314,022	1,303	37.98	90.96
#23	F_8	4,266,541	1,288	37.92	90.76
#24	F_8	3,394,724	1,025	37.81	90.36
#25	F_8	4,143,484	1,251	38.03	91.15
#26	F_8	4,622,780	1,396	37.96	90.89
#27	F_8	4,431,177	1,338	37.98	90.94
#28	F_8	4,116,516	1,243	37.82	90.42
#29	F_8	4,031,111	1,217	37.96	90.91
#30	F_7	4,136,678	1,249	37.9	90.7

Supplementary Table S1 (*Continued*)

RILs	Generation	Reads	Yield (Mbp)	Mean Quality Score	% Bases ≥ 30
#32	F_8	4,365,737	1,318	37.96	90.89
#33	F_8	4,136,941	1,249	37.89	90.66
#34	F_8	3,782,524	1,142	37.87	90.6
#35	F_8	4,421,972	1,335	37.94	90.85
#36	F_8	4,120,020	1,244	37.91	90.91
#37	F_8	4,235,372	1,279	37.88	90.6
#38	F_8	3,524,514	1,064	37.91	90.75
#40	F_7	4,311,849	1,302	37.8	90.33
#41	F_6	4,037,109	1,219	37.87	90.6
#42	F_8	3,845,607	1,161	37.88	90.64
#43	F_8	4,040,351	1,220	37.89	90.66
#44	F_8	4,203,640	1,269	37.92	90.76
#45	F_8	4,135,601	1,249	37.9	90.7
#46	F_8	4,492,003	1,357	37.9	90.68
#47	F_8	4,428,983	1,338	37.91	90.7
#48	F_8	4,267,012	1,289	37.91	90.71
#49	F_8	3,870,413	1,169	37.93	90.78
#50	F_8	4,063,327	1,227	37.8	90.37
#51	F_8	4,009,906	1,211	37.88	90.59
#52	F_8	4,151,808	1,254	37.88	90.6
#53	F_8	3,956,139	1,195	37.93	90.78
#54	F_8	3,947,970	1,192	37.93	90.81
#55	F_8	3,745,973	1,131	37.85	90.53
#56	F_8	4,520,515	1,365	37.86	90.53
#57	F_8	3,981,182	1,202	37.91	90.73
#58	F_8	3,276,567	990	37.84	90.45
#59	F_8	4,006,024	1,210	37.9	90.68
#60	F_8	4,072,918	1,230	37.84	90.48
#61	F_8	4,124,380	1,246	37.92	90.76
#62	F_8	4,293,082	1,297	37.9	90.71

Supplementary Table S1 (*Continued*)

RILs	Generation	Reads	Yield (Mbp)	Mean Quality Score	% Bases ≥ 30
#63	F_8	4,094,581	1,237	37.87	90.6
#64	F_6	3,824,317	1,155	37.9	90.7
#65	F_8	3,665,910	1,107	37.81	90.38
#66	F_8	3,908,914	1,180	37.87	90.57
#67	F_8	3,482,369	1,052	37.86	90.56
#68	F_8	3,833,484	1,158	37.89	90.64
#69	F_8	3,907,290	1,180	37.89	90.67
#70	F_8	3,936,813	1,189	37.9	90.67
#71	F_8	4,424,447	1,336	37.91	90.72
#72	F_8	3,651,879	1,103	37.87	90.58
#73	F_8	3,657,706	1,105	37.9	90.69
#74	F_7	3,562,671	1,076	37.88	90.62
#75	F_8	3,765,068	1,137	37.89	90.66
#76	F_8	3,635,603	1,098	37.86	90.54
#77	F_8	4,344,257	1,312	37.97	90.92
#78	F_8	3,879,087	1,171	37.79	90.3
#79	F_8	3,848,935	1,162	37.81	90.37
#80	F_8	3,693,964	1,116	37.97	90.93
#81	F_8	3,827,518	1,156	37.95	90.85
#82	F_8	2,928,960	885	37.92	90.75
#83	F_8	4,165,399	1,258	37.91	90.73
#84	F_8	4,282,741	1,293	38.03	91.14
#85	F_8	4,132,778	1,248	37.86	90.55
#86	F_8	3,978,355	1,201	37.93	90.79
#87	F_8	4,105,441	1,240	37.81	90.38
#88	F_8	3,955,291	1,194	37.93	90.79
#89	F_7	4,137,078	1,249	37.96	90.9
#90	F_8	3,692,210	1,115	37.81	90.36
#91	F_8	4,586,491	1,385	37.95	90.86
#92	F_8	4,329,723	1,308	37.91	90.73

Supplementary Table S1 (*Continued*)

RILs	Generation	Reads	Yield (Mbp)	Mean Quality Score	% Bases ≥ 30
#93	F_8	4,034,163	1,218	37.93	90.8
#94	F_8	3,907,078	1,180	37.96	90.87
#95	F_8	4,012,638	1,212	37.95	90.87
#96	F_8	3,985,819	1,204	37.87	90.58
#97	F_8	3,580,708	1,081	37.89	90.68
#98	F_8	3,504,329	1,058	37.81	90.38
PI499262	-	4,502,928	1,360	37.91	90.68
PI499262	-	4,536,678	1,370	37.97	90.92
PI499262	-	4,813,317	1,454	37.85	90.51
PI499262	-	4,257,763	1,286	37.91	90.71
KU-2078	-	4,027,869	1,216	37.93	90.82
KU-2078	-	4,256,860	1,286	37.89	90.69
KU-2078	-	4,093,295	1,236	37.87	90.6
KU-2078	-	4,313,393	1,303	37.88	90.63
Average	-	4,070,273	1,229	37.90	90.68
Median	-	4,102,203	1,239	37.90	90.69
Minimum	-	2,928,960	885	37.79	90.30
Maximum	-	4,813,317	1,454	38.03	91.15

Supplementary Table S2 GRAS-Di marker quality and corresponding parental genotypes. The makers are ranked using A, B, C, D and E in descending order of quality.

Quality	All markers	Polymorphic in the parental lines	Monomorphic in the parental lines but polymorphic in the RILs
A	20,389	18,656	1,733
B	12,758	12,123	635
C	18,759	17,681	1,078
D	25,094	23,095	1,999
E	1,198	1,126	72
ABCDE	78,198	72,681	5,517
ABCD	77,000	71,555	5,445
ABC	51,906	48,460	3,446

Supplementary Table S3 Summary of the number of GRAS-Di markers (per Mbp) anchored to the *Ae. tauschii* D genome. The number of markers per Mbp is shown in parentheses.

Category	All	F-R distance < 300
1D	3,805<br>(7.57)	3,761<br>(7.49)
2D	5,034<br>(7.72)	4,974<br>(7.63)
3D	4,879<br>(7.78)	4,797<br>(7.65)
4D	4,445<br>(8.45)	4,380<br>(8.33)
5D	4,188<br>(7.25)	4,126<br>(7.15)
6D	3,570<br>(7.20)	3,493<br>(7.04)
7D	4,693<br>(7.28)	4,623<br>(7.17)
Total	30,614<br>(7.61)	30,154<br>(7.49)

Supplementary Table S4 Summary of the number of loci in each linkage map.

Marker set name	tau-D qABC	tau-D qAB	tau-D qA	qAB	qA
LG1	575	367	269	435	318
LG2	852	552	398	683	486
LG3	740	466	331	586	417
LG4	432	241	175	301	221
LG5	761	526	399	662	497
LG6	445	269	202	342	245
LG7	716	475	341	587	424
total	4521	2896	2115	3596	2608
No. loci/cM	0.85	0.68	0.79	0.96	0.87
No. loci/Mbp	1.12	0.72	0.53	0.53	0.52
Suggested LOD	7.44	7.06	6.79	7.24	6.97

Supplementary Table S5 Summary of linkage map length (cM) and log-likelihood for five marker sets.

Marker set name	tau-D qABC	tau-D qAB	tau-D qA	qAB	qA
LG1	595.14	395.27	323.22	592.28	480.73
LG2	968.77	1,389.41	465.41	737.41	523.07
LG3	790.75	615.26	417.04	442.19	343.9
LG4	532.6	271.12	308.78	604.01	493.54
LG5	817.49	564.72	432.65	365.26	359.81
LG6	473.4	367.15	308.32	377.42	300
LG7	1,137.42	643.47	412.62	639.25	509.17
Total	5,315.57	4,246.4	2,668.04	3,757.82	3,010.22
Log-likelihood	-41,629.2	-27,430.4	-21,371.7	-30,162.6	-25,011.2

Supplementary Table S6 Summary of genetic variance, environment variance, variance of genotype by environment, and heritability for the 18 traits in the RILs of *Aegilops tauschii*

Traits	V_g	V_e	$V_{g\,x\,e}$	V_r	*h*
HD	52.808	33.834	1.639	16.825	0.50
FT	47.657	33.100	1.754	17.714	0.48
SL	1.386	0.600	0.458	0.613	0.45
NSp	1.777	0.316	0.614	0.422	0.57
SpD	0.016	0.002	0.003	0.006	0.58
NISp	0.332	0.000	0.258	0.323	0.36
TAL	0.167	0.023	0.061	0.066	0.53
MAL	0.110	0.001	0.068	0.067	0.45
SpL	0.446	0.109	0.075	0.159	0.56
SpW	0.076	0.048	0.019	0.052	0.39
SpLWr	0.078	0.007	0.020	0.019	0.63
EGL	0.167	0.032	0.056	0.069	0.52
EGW	0.069	0.039	0.022	0.035	0.42
EGLWr	0.050	0.008	0.016	0.012	0.58
GH	0.010	0.018	0.018	0.016	0.16
GL	0.109	0.357	0.112	0.128	0.15
GW	0.044	0.046	0.030	0.031	0.29
GLWr	0.024	0.003	0.053	0.005	0.28

V_g: genetic variance; V_e: environmental variance; $V_{g\,x\,e}$: variance of genotype by environment interaction; V_r : residual variance; *h*: heritability

The values for SL were calculated after logarithmic transformation of the trait values.

Supplementary Table S7 Thresholds of the LOD scores in the QTL analyses.

Season	2017-2018		2018-2019	
Significance level	5%	10%	5%	10%
HD	6.99	6.48	7.07	6.38
FT	7.20	6.51	7.05	6.52
SL	7.15	6.51	7.24	6.55
NSp	7.19	6.51	7.24	6.40
SpD	7.45	6.80	7.62	6.84
TAL	7.70	6.80	7.04	6.47
MAL	7.75	6.82	7.40	6.65
SpL	7.35	6.63	7.22	6.63
SpW	7.27	6.61	7.10	6.58
SpLWr	7.33	6.66	7.63	6.87
EGL	7.18	6.62	7.53	6.70
EGW	7.49	6.82	7.45	6.77
EGLWr	7.36	6.65	7.34	6.56
GL	6.88	6.42	7.18	6.58
GW	7.03	6.48	7.32	6.64
GLWr	7.33	6.69	7.32	6.68
GH	7.31	6.61	7.67	6.92

Supplementary Table S8 List of candidate genes located in the identified QTL regions.

Gene ID	Chr.	Position (bp)	Gene	QTL markers near genes 2017-2018 / 2018-2019 (bp)	Positions 2017-2018 / 2018-2019	Traits
AET3Gv20300900	3	102,944,017	WAG-2g MADS box transcription factor [*Aegilops tauschii*]	AMP0350288 / AMP0031578	128,208,785 / 114,361,083	FT
AET3Gv20308400	3	107,936,489	cytokinin dehydrogenase 2-like [*Aegilops tauschii*]			
AET3Gv20309400	3	108,960,945	cytokinin dehydrogenase 2-like [*Aegilops tauschii* subsp. *tauschii*]			
AET3Gv20310600	3	109,875,028	protein RICE FLOWERING LOCUS T 1-like [*Aegilops tauschii* subsp. *tauschii*]			
AET3Gv20316100	3	113,386,833	transcription factor PCF5-like [*Aegilops tauschii* subsp. *tauschii*]			
AET7Gv20290900	7	69,247,183	protein HEADING DATE 3A (VRN3/FT) [*Aegilops tauschii* subsp. *tauschii*]	AMP0099995 / AMP0099995	69,293,840 / 69,293,840	FT
AET7Gv20333700	7	84,554,513	zinc finger protein CONSTANS-LIKE 2-like [*Aegilops tauschii* subsp. *tauschii*]			
AET7Gv221091300	7	562,206,136	MADS box transcription factor	AMP0063209 / AMP0252067	557,067,506 / 560,772,615	EGLWr
AET7Gv21207900	7	598,963,794	AP2-like ethylene-responsive transcription factor AIL5 [*Aegilops tauschii* subsp. *tauschii*]	AMP0092717 / AMP0092717	599,236,655 / 599,236,655	GLWr

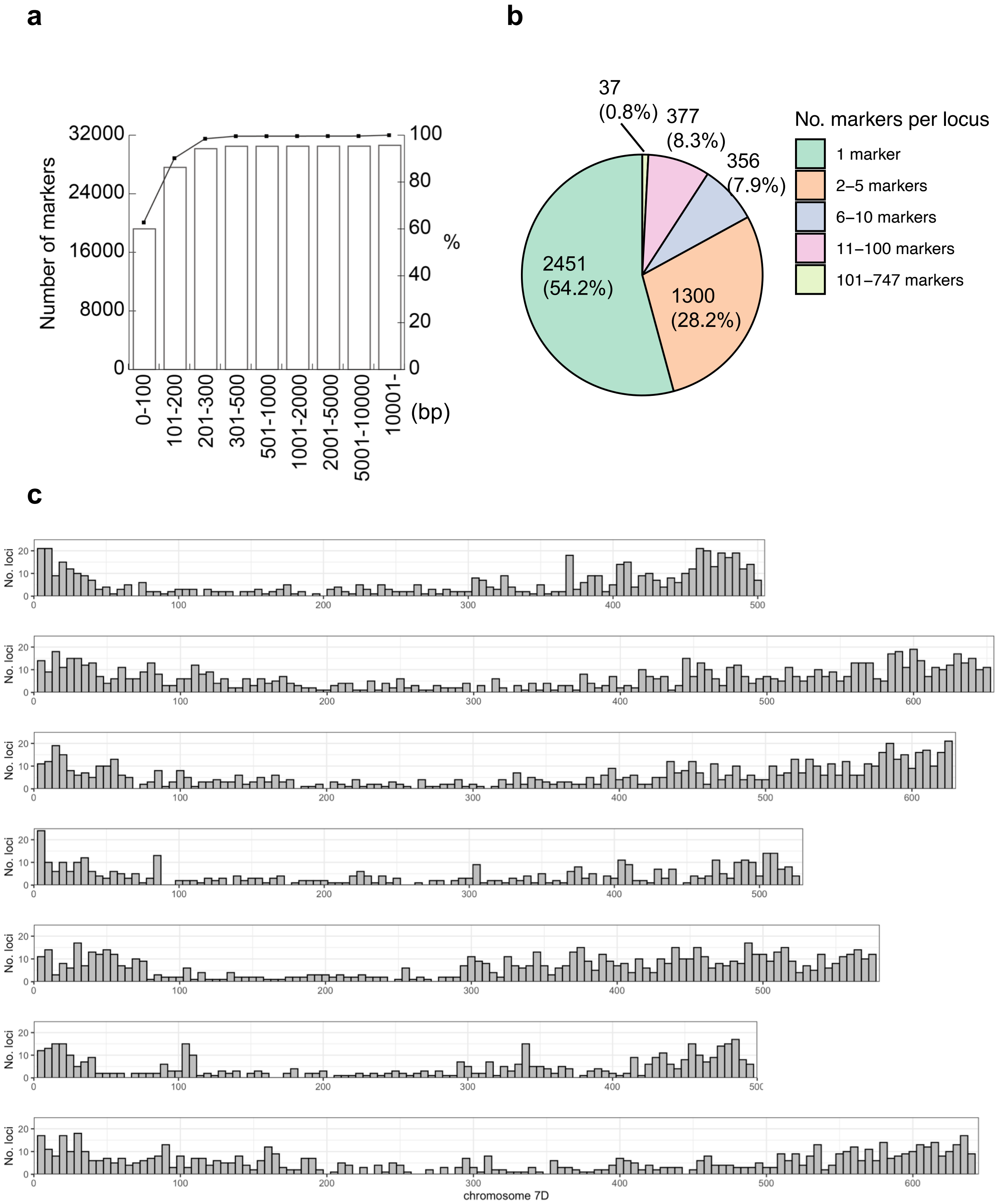

Supplementary Fig. S1 GRAS-Di markers and segregating loci in the *Ae. tauschii* KU-2078/PI499262 RILs. (a) Cumulative distribution of physical distance between forward and reverse reads aligned to the reference genome of *Ae. tauschii*. (b) Pie chart shows the percentage of GRAS-Di markers per locus. (c) Distribution of loci on the chromosomes of *Ae. tauschii*. The density of loci increased towards the end of the chromosomes. The number of loci per 5 Mbp is shown in each bin. The bar plot was created with the R package ggplot2 (Wickham 2016).

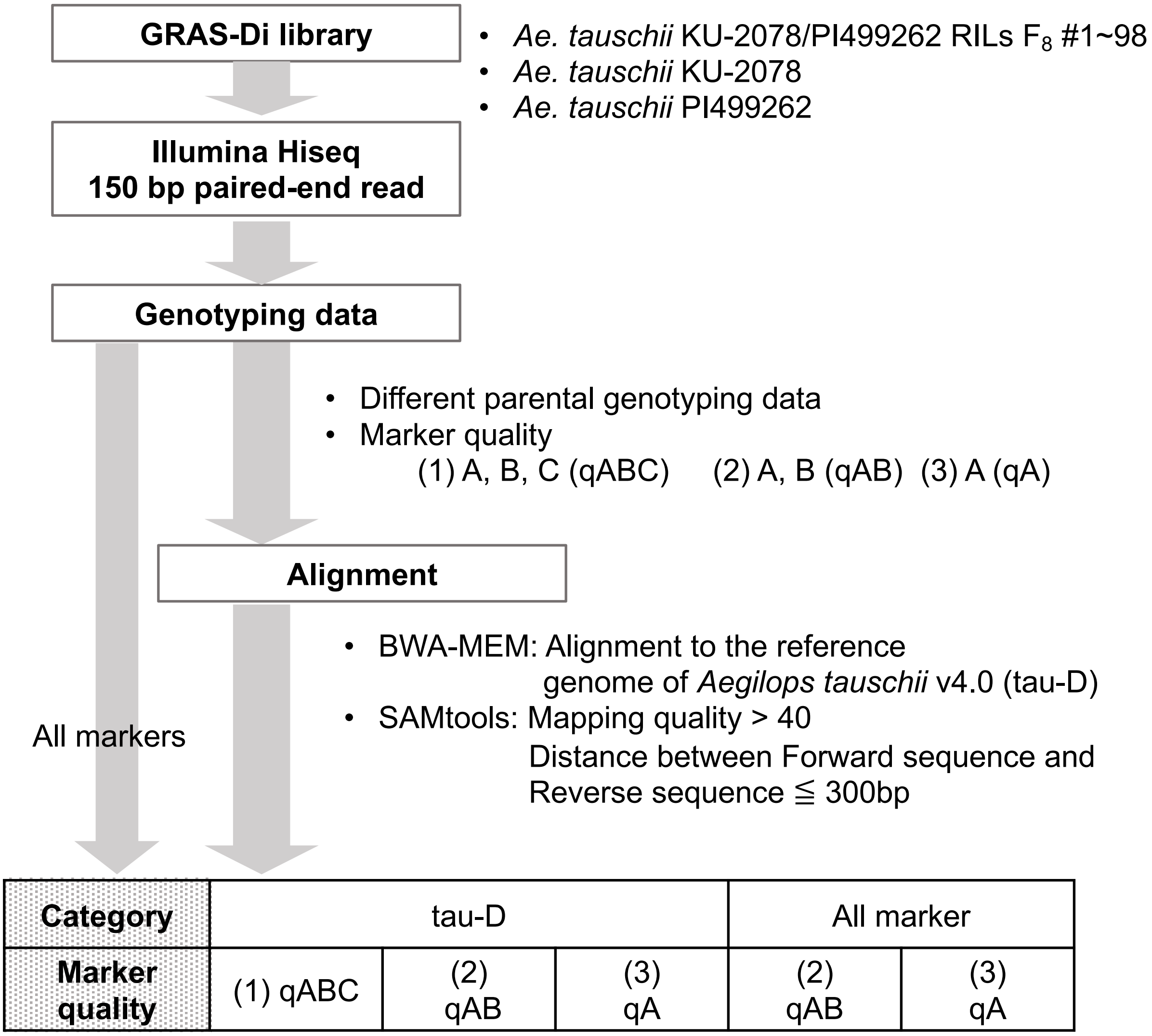

Supplementary Fig. S2 Workflow of the selection method used for GRAS-Di markers.

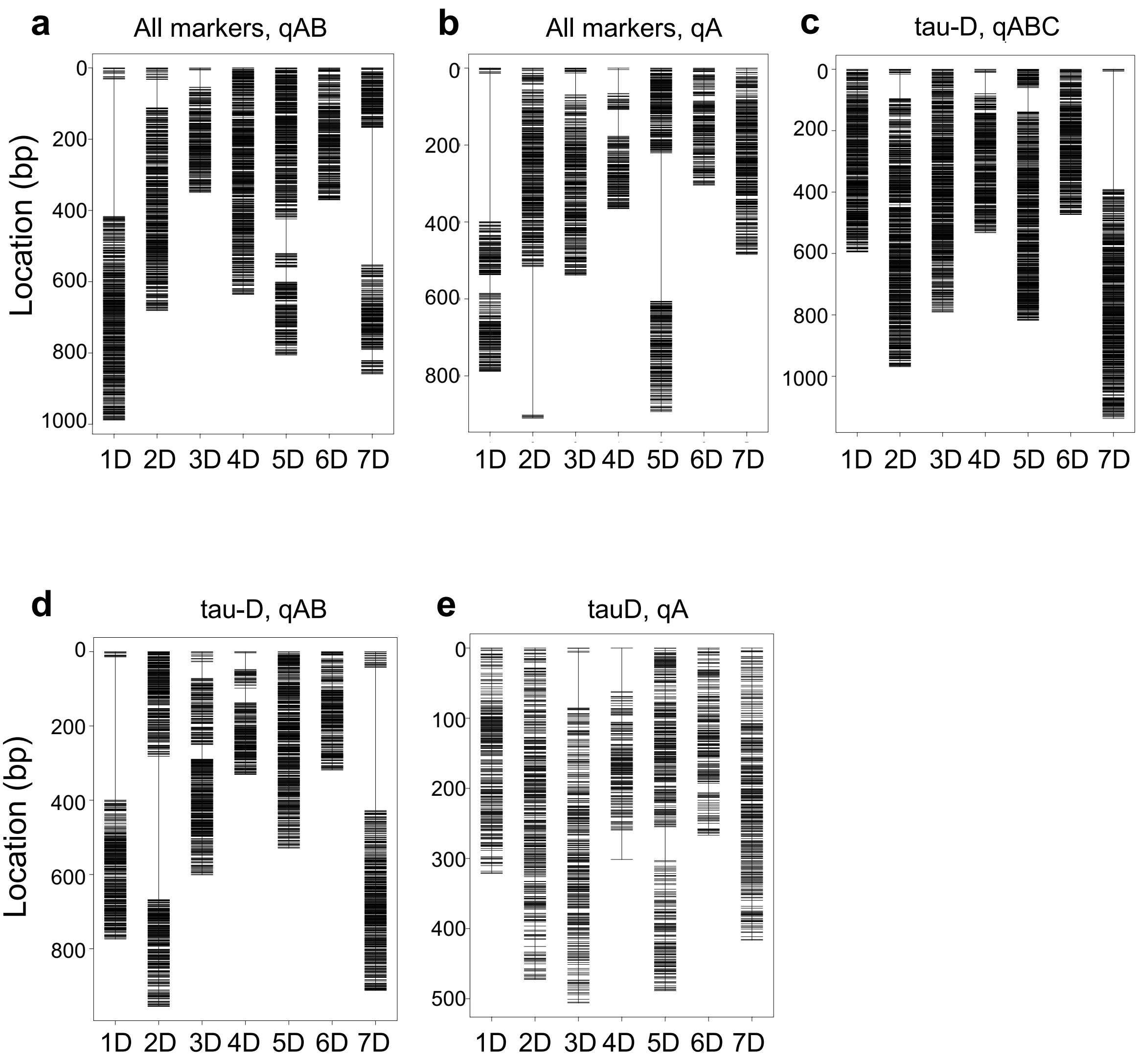

Supplementary Fig. S3 High-density linkage maps of the *Ae. tauschii* KU-2078/PI499262 RILs. The x-axis represents the linkage group number, and the y-axis indicates the genetic distance (cM) in each linkage group. (a) All markers of quality A or B were used. (b) All markers of quality A were used. (c) The markers of quality A, B or C that were mapped to *Ae. tauschii* genome sequences were used. (d) The markers of quality A or B that were mapped to *Ae. tauschii* genome sequences were used. (e) The markers of quality A that were mapped to *Ae. tauschii* genome sequences were used. The linkage maps were generated with the R package R/qTL (Broman et al. 2003).

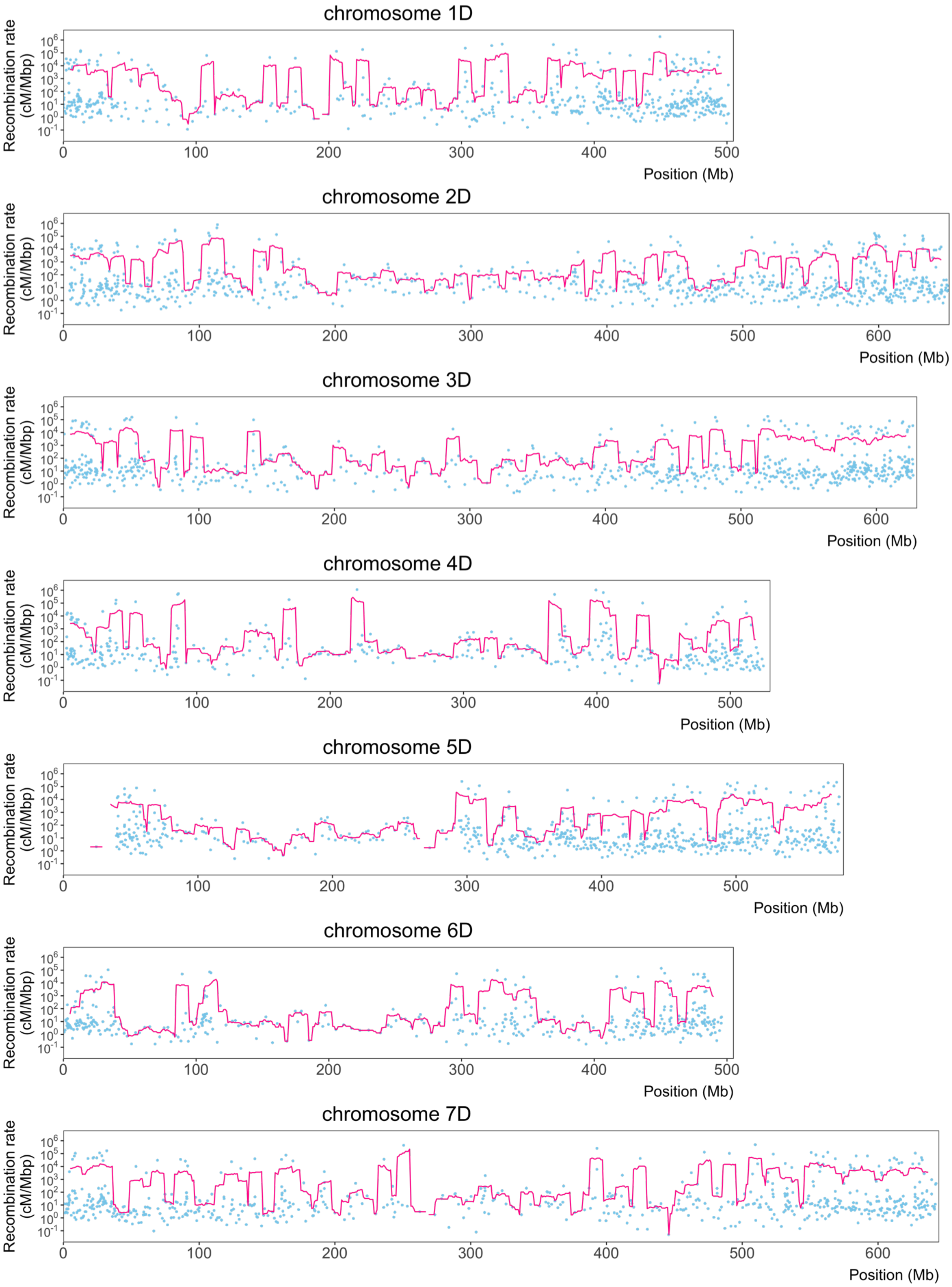

Supplementary Fig. S4 Distribution of the recombination rate (cM/Mbp) over the chromosomes in the *Ae. tauschii* KU-2078/PI499262 RILs. The y-axis represents the recombination rate (cM/Mb), and the x-axis indicates positions on the physical map of *Ae. tauschii*. The window size is 10 Mbp, and the step size is one mega-base pair. The modified tau-D qABC linkage map was used to estimate the recombination rate.

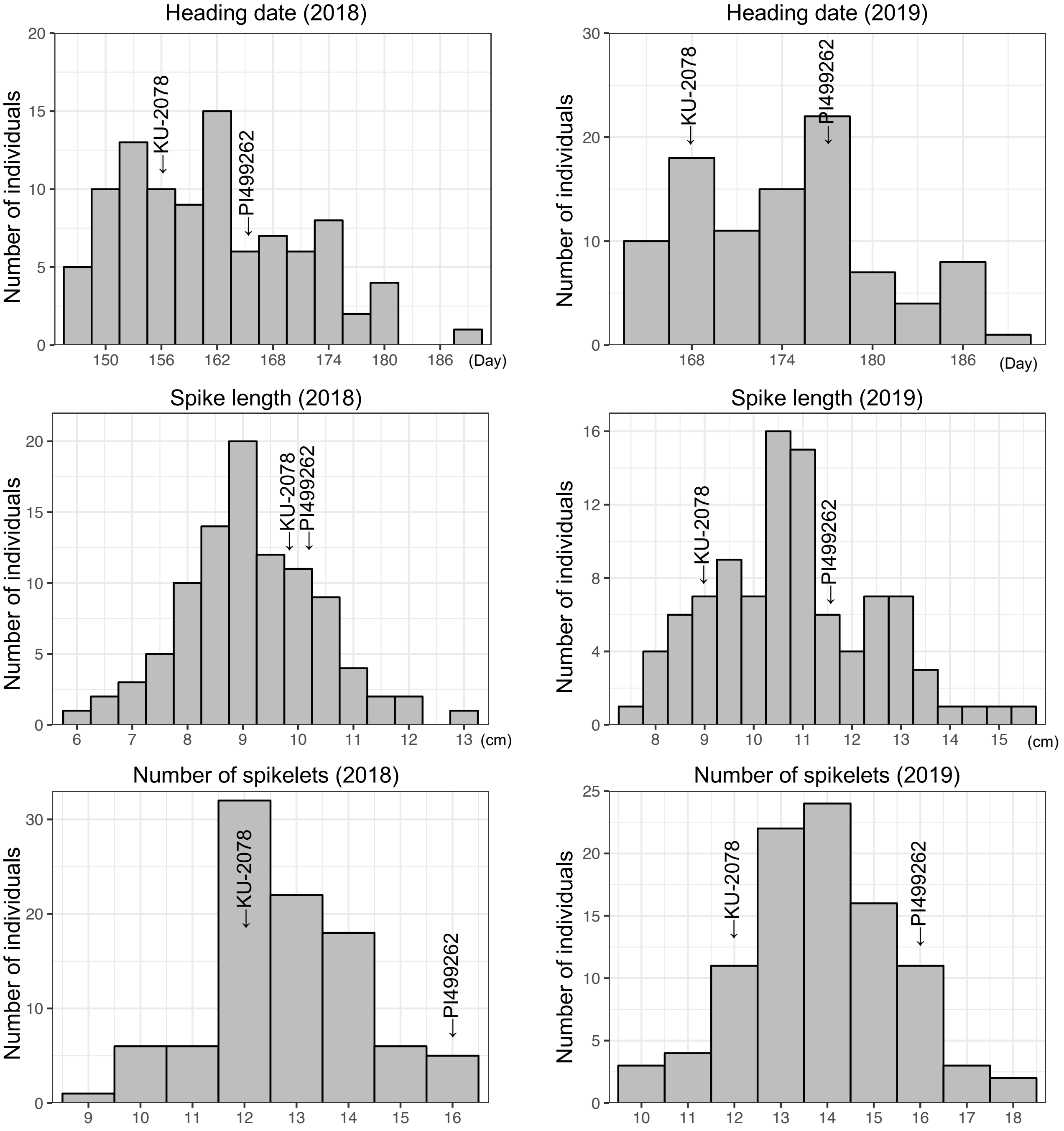

Supplementary Fig. S5 Histograms of heading date and spike morphology-related traits in *Ae. tauschii* KU-2078/PI499262 RILs in the two growth seasons. The histograms were created with the R package ggplot2 (Wickham 2016).

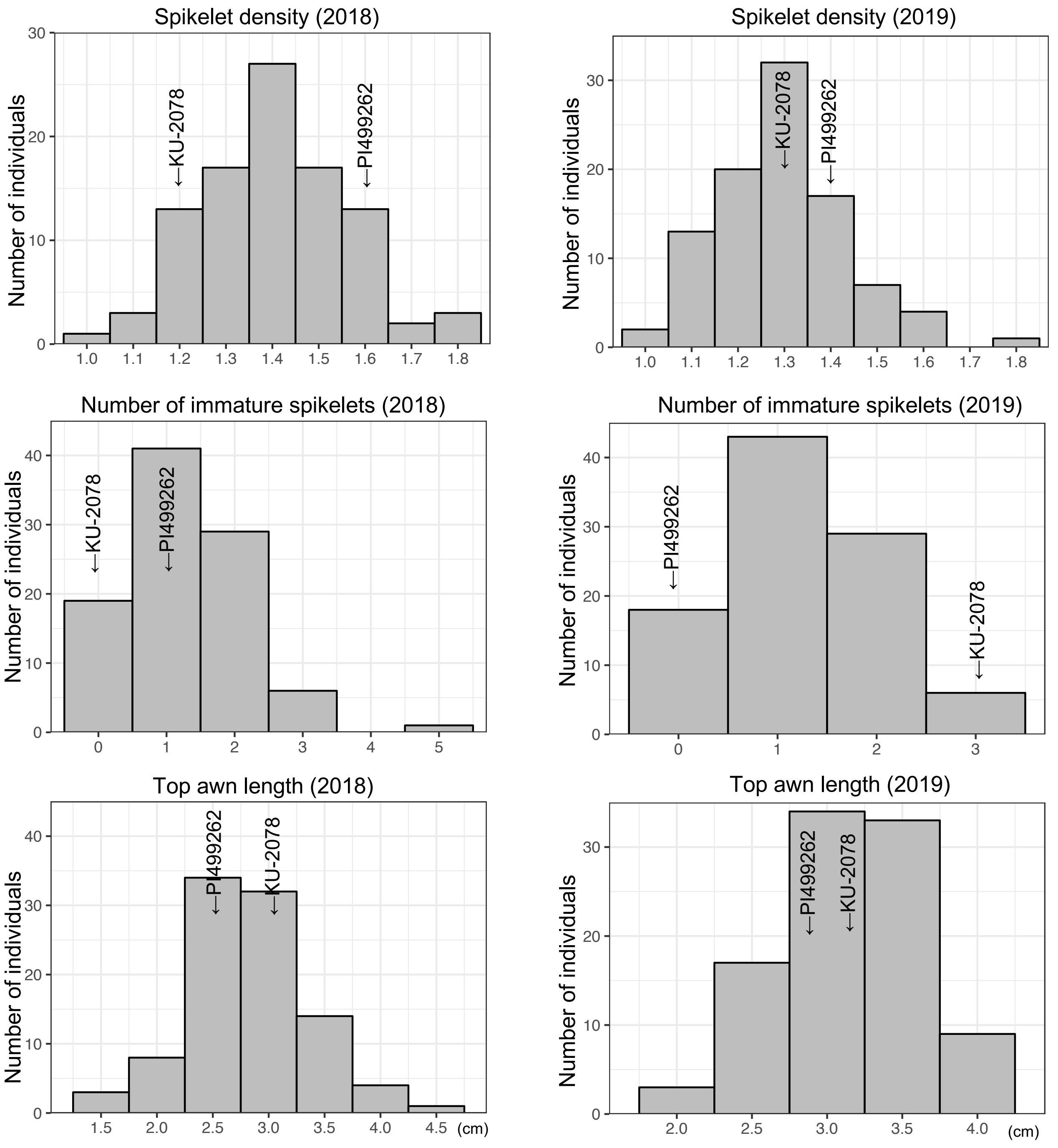

Supplementary Fig. S5 (*Continued*)

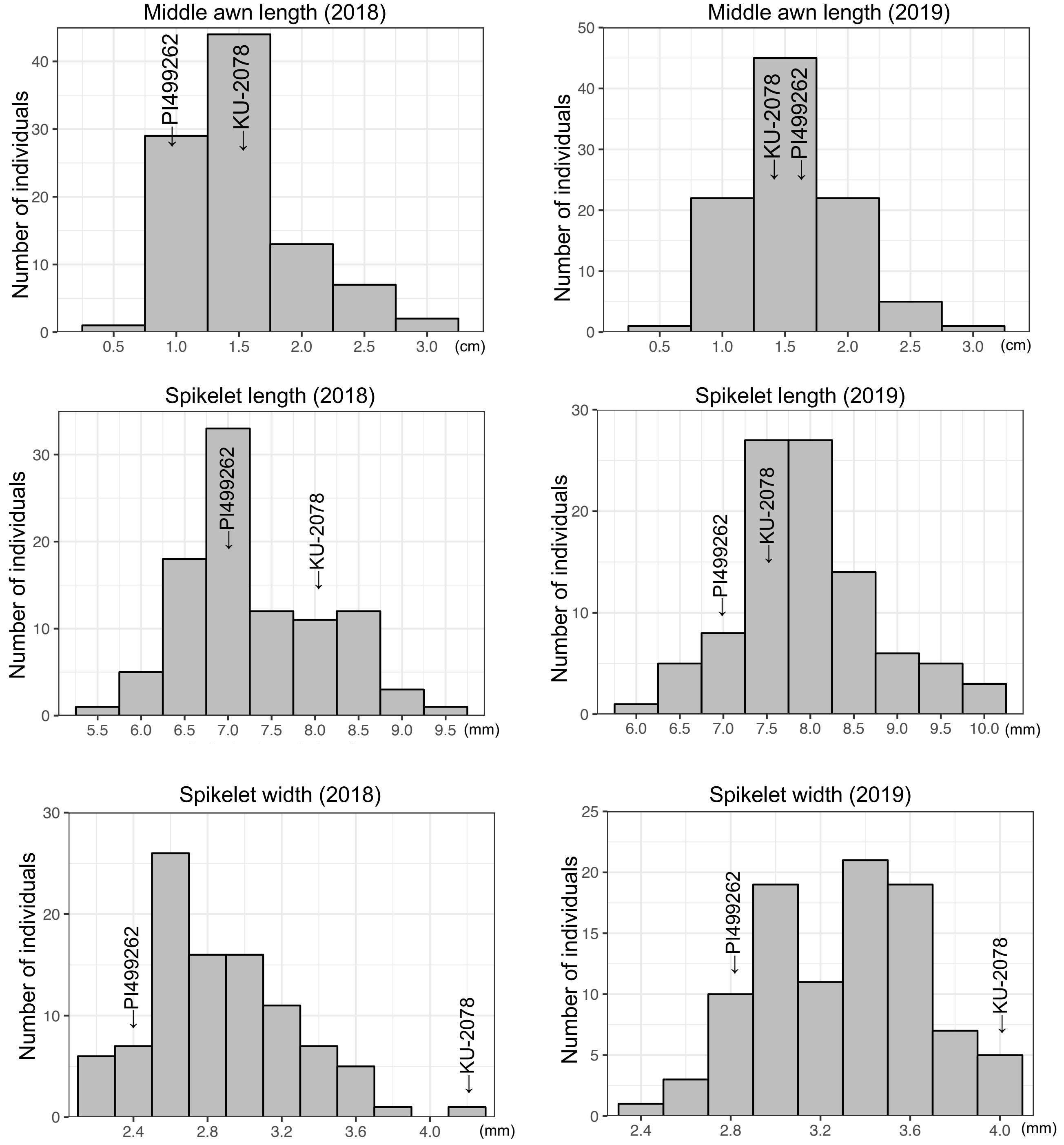

Supplementary Fig. S5 (*Continued*)

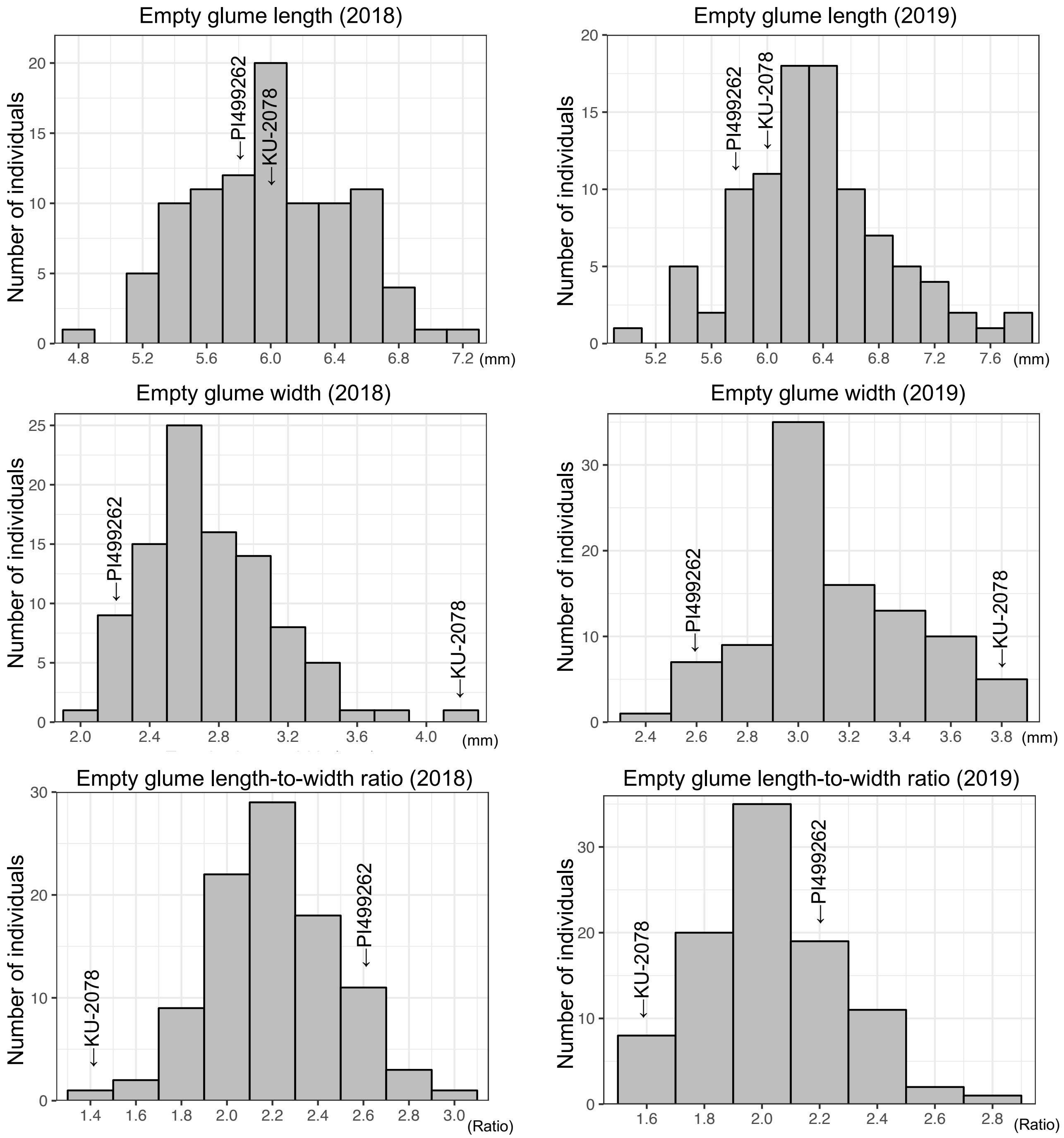

Supplementary Fig. S5 (*Continued*)

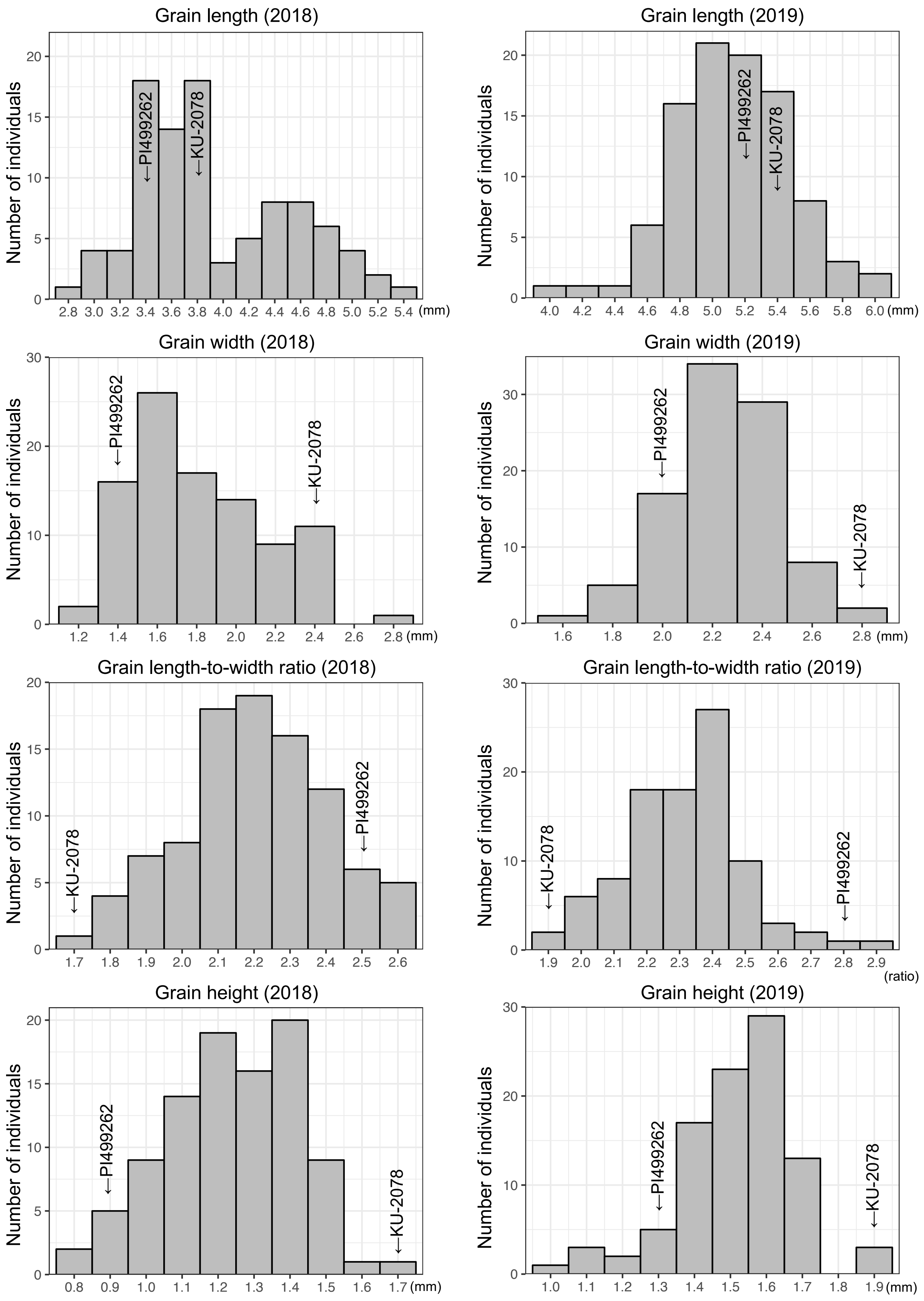

Supplementary Fig. S5 (*Continued*)

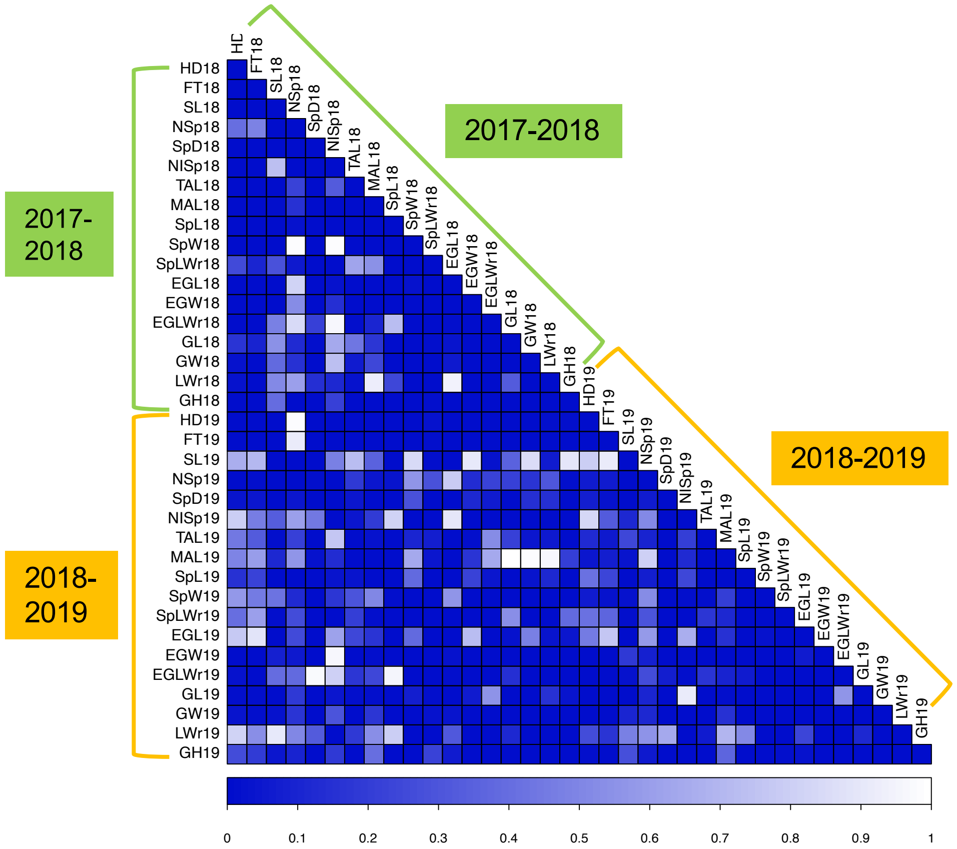

Supplementary Fig. S6 Pearson's correlation coefficients and corresponding P-values between the traits examined in the *Ae. tauschii* KU-2078/PI499262 RILs in seasons 2017-2018 and 2018-2019. The heatmap was created with the R package corrplot (Wei and Simko 2017).

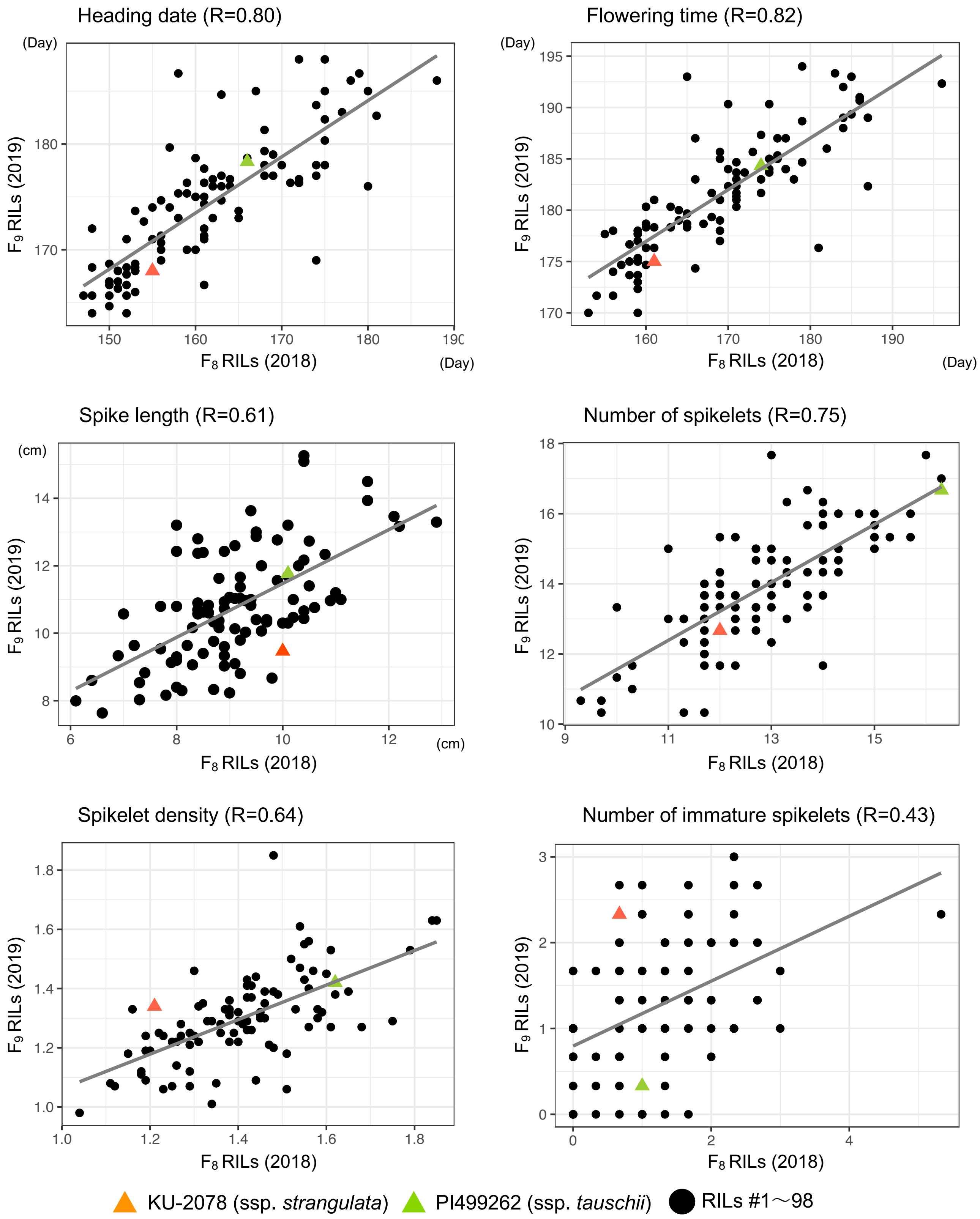

Supplementary Fig. S7 Scatter plots showing the correlation between each pair of traits in the *Ae. tauschii* KU-2078/PI499262 RILs in seasons 2017-2018 and 2018-2019. The plots were created with the R package ggplot2 (Wickham 2016).

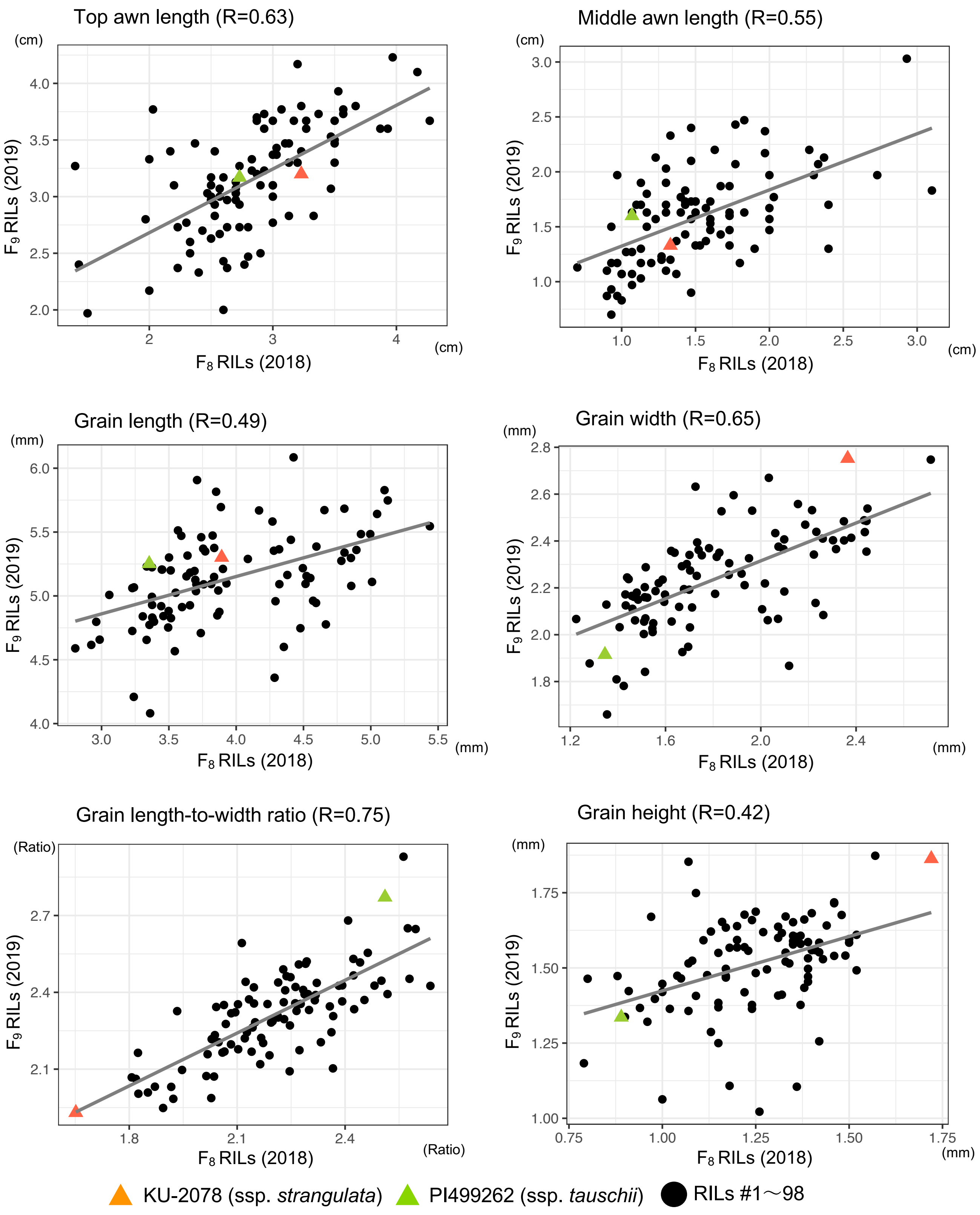

Supplementary Fig. S7 (*Continued*)

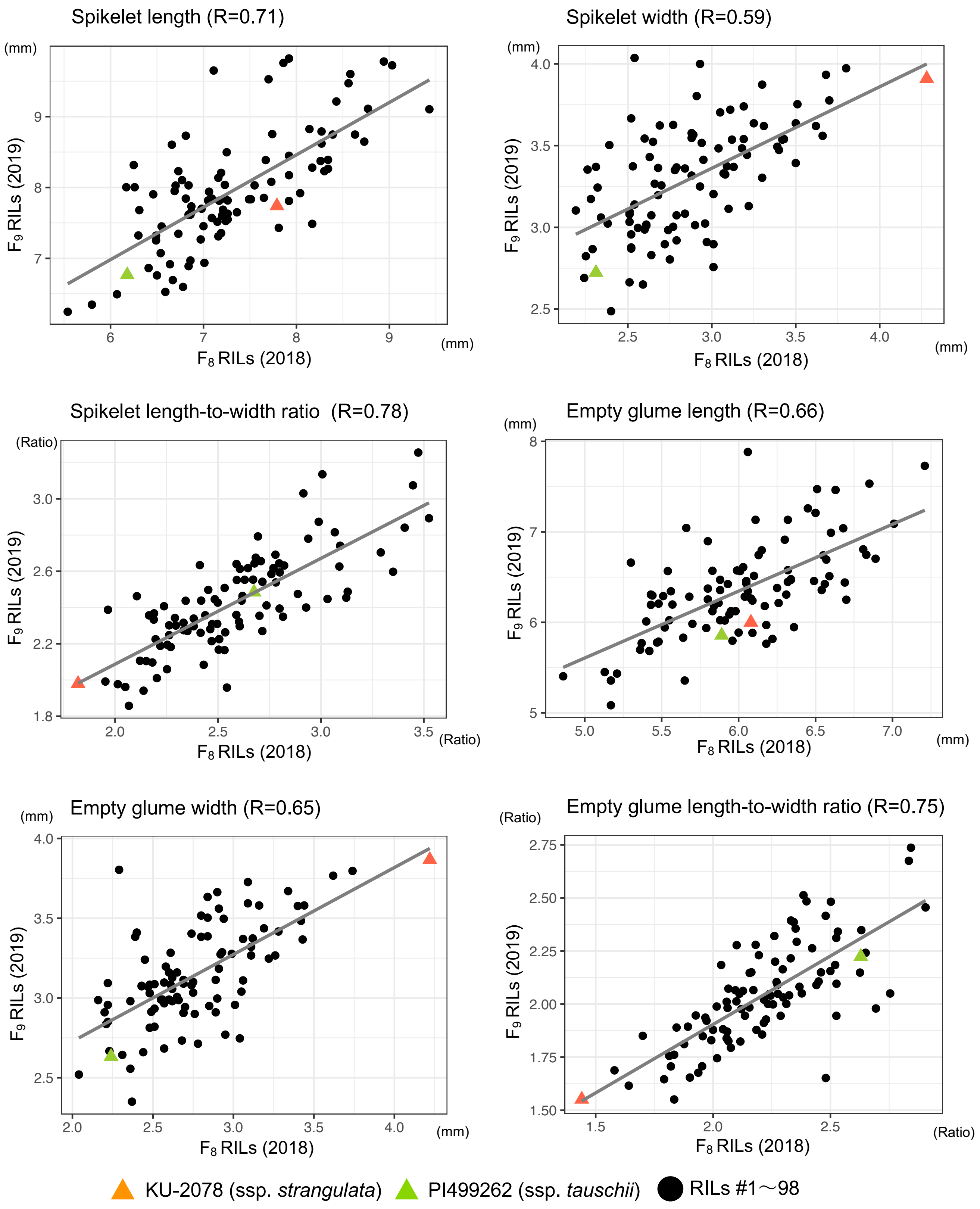

Supplementary Fig. S7 (*Continued*)

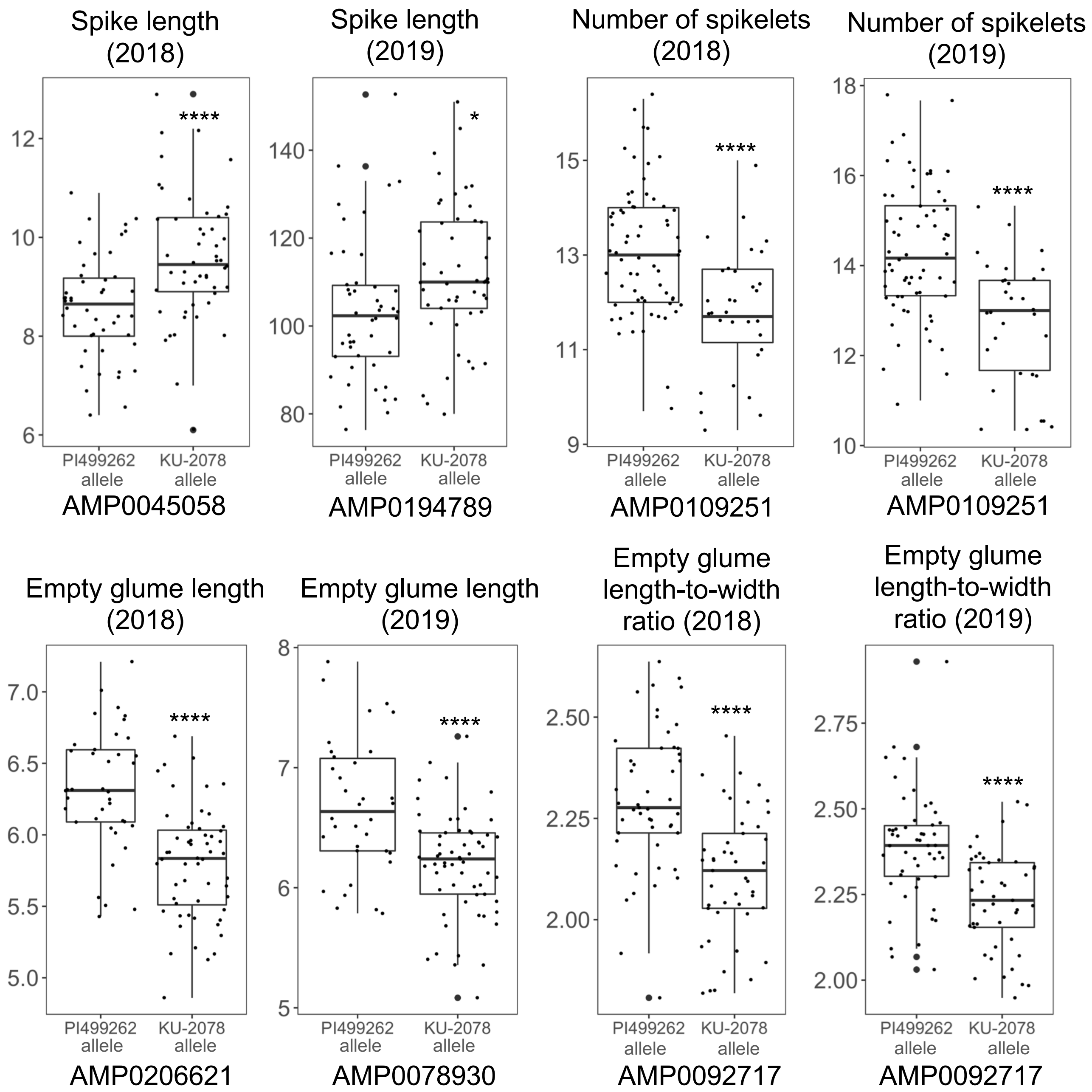

Supplementary Fig. S8 Box and dot plots comparing each trait between the two different alleles at the QTL-linked markers in the *Ae. tauschii* KU-2078/PI499262 RIL F_8 (2018)/F_9 (2019) generations. Welch's two-sample t-test was performed to assess statistical significance between two alleles (*P<0.05, **P<0.01, ***P<0.005, ****P< 0.001). The box plots were created with the R package ggplot2 (Wickham 2016).

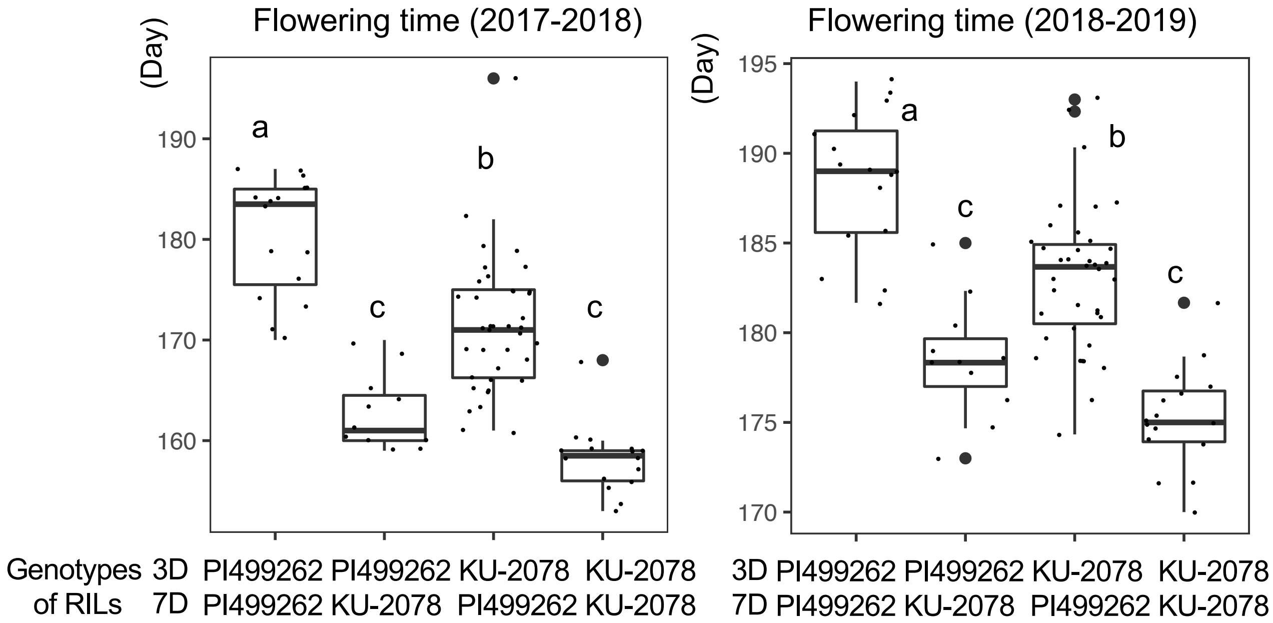

Supplementary Fig. S9 Box and dot plots for flowering time in pairwise combinations of the two different genotypes of RILs at the QTL-linked markers in the *Ae. tauschii* KU-2078/PI499262 RIL F_8 (2018)/F_9 (2019) generation. The same letters indicate no significant difference (P > 0.05, Tukey-Kramer HSD test). The box plots were created with the R package ggplot2 (Wickham 2016).

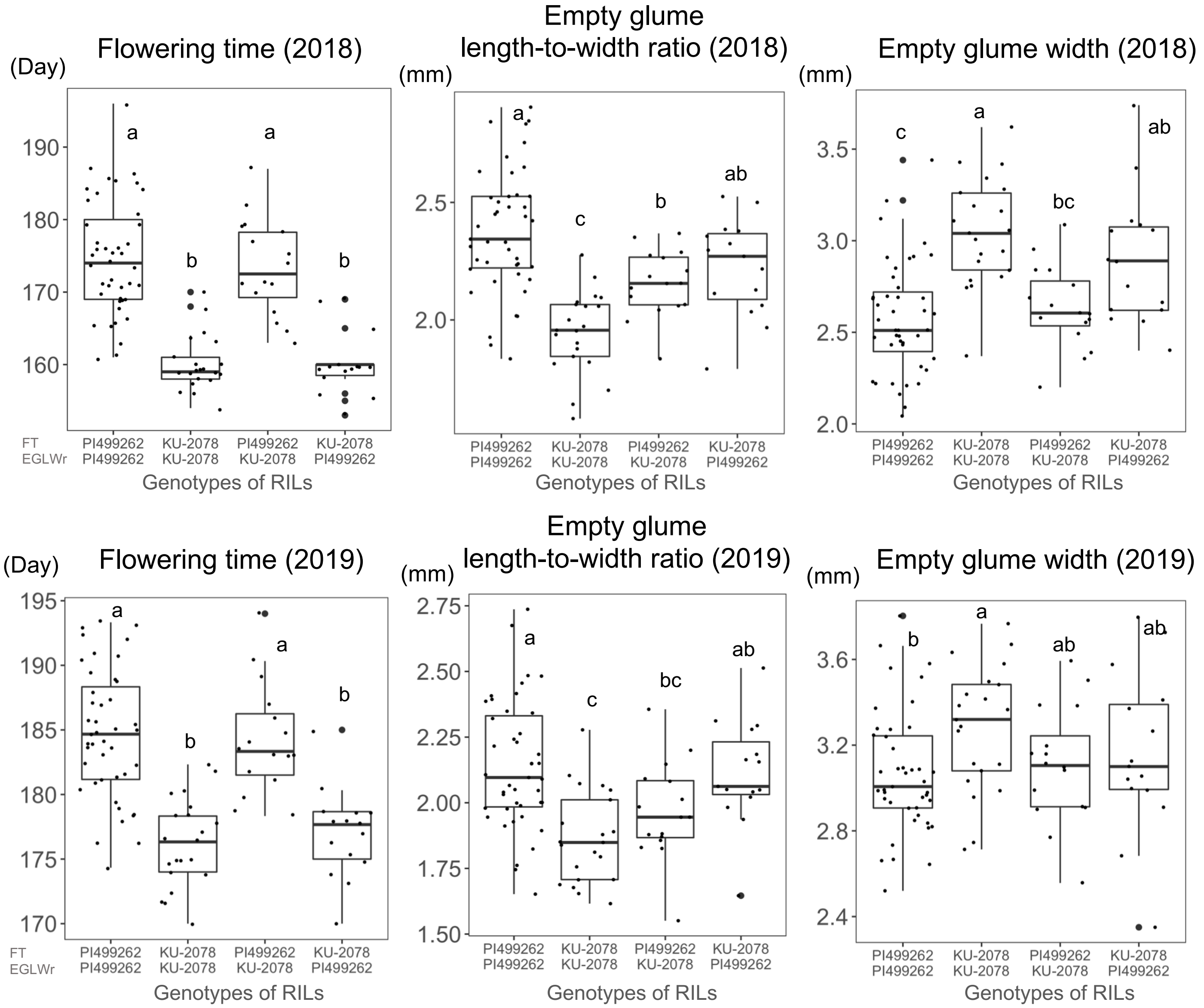

Supplementary Fig. S10 Box and dot plots for each trait in pairwise combinations of the two different genotypes (PI499262 allele and KU-2078 allele) at the markers linked to 7D QTLs of flowering time, empty glume length-to-width ratio and empty glume width in the *Ae. tauschii* KU-2078/PI499262 RILs. The same letters indicate no significant difference (P > 0.05, Tukey-Kramer HSD test). The box plots were created with the R package ggplot2 (Wickham 2016).

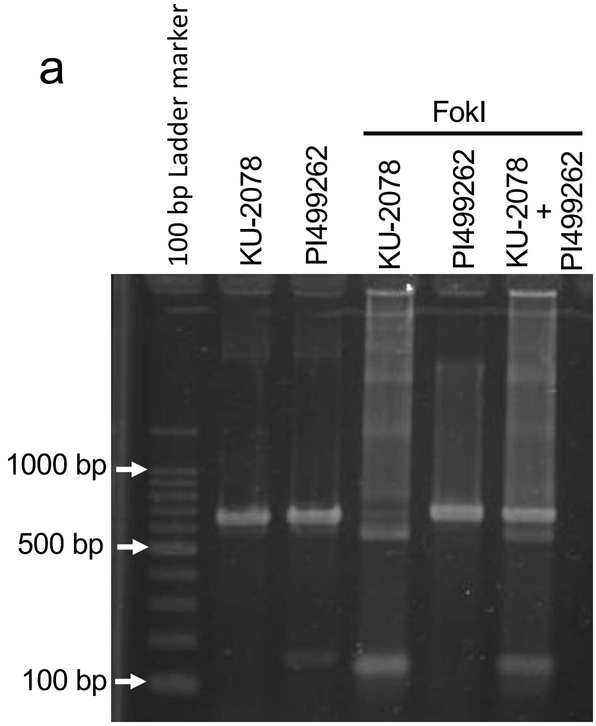

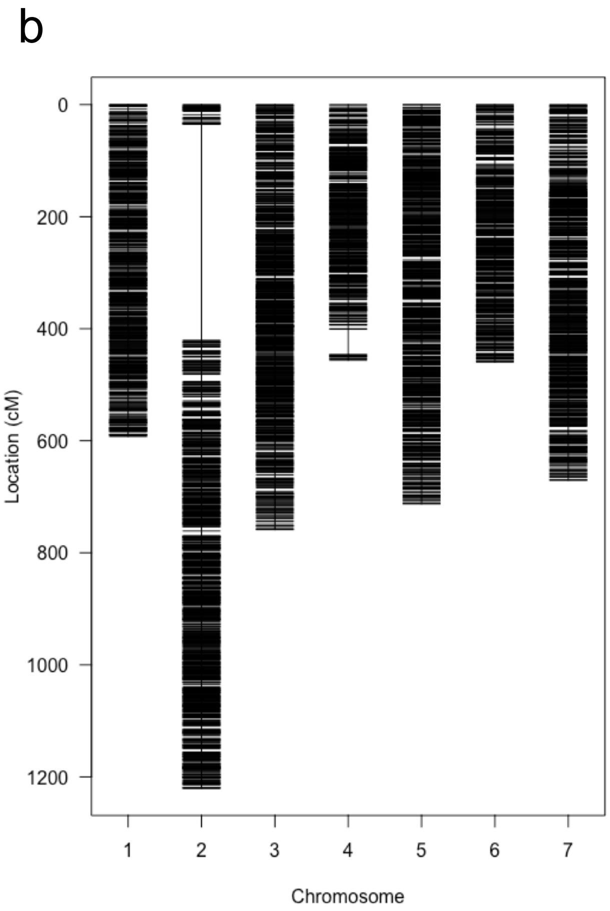

Supplementary Fig. S11 Linkage map including *FT*/*VRN3* allele information. (a) CAPS marker for the 67th nucleotide substitution in *FT*/*VRN3* between the parental accessions KU-2078 and PI499262. The 0.6 kb PCR fragment was digested with FokI. The PCR product from KU-2078 was separated into two fragments. (b) Linkage map constructed based on the tau-D qABC GRAS-Di marker set and CAPS marker of *FT*/*VRN3* after removing three heterozygous RILs. The linkage map was generated with the R package R/qTL (Broman et al. 2003).
